# A DOF transcription factor GLW9/OsDOF25 regulates grain shape and tiller angle in rice

**DOI:** 10.1111/pbi.70064

**Published:** 2025-03-22

**Authors:** Huan Shi, Pingbo Li, Peng Yun, Yun Zhu, Hao Zhou, Lu Wang, Bian Wu, Yipei Wang, Guangming Lou, Qin Huang, Guanjun Gao, Qinglu Zhang, Junxiao Chen, Jinbo Li, Jinghua Xiao, Aiqing You, Yuqing He

**Affiliations:** ^1^ National Key Laboratory of Crop Genetic Improvement, Hubei Hongshan Laboratory Huazhong Agricultural University Wuhan China; ^2^ Institute of Food Crop Hubei Academy of Agricultural Science Wuhan China; ^3^ Institute of Wetland Agriculture and Ecology Shandong Academy of Agricultural Sciences Jinan China; ^4^ Rice Research Institute Anhui Academy of Agricultural Sciences Hefei China; ^5^ State Key Laboratory of Crop Gene Exploration and Utilization in Southwest China, Rice Research Institute Sichuan Agricultural University Chengdu China

**Keywords:** rice, *GLW9*, grain shape, *EXPA6*, tiller angle, *OsPIN1b*

## Abstract

Grain shape and tiller angle are two important agronomic traits influencing grain yield and quality in rice. Herein, we map‐based cloned a grain shape gene *GLW9* (*Grain Length and Width on chromosome 9*), which encodes a DNA binding with one finger (DOF) family transcription factor OsDOF25. *GLW9* positively regulates grain length and negatively regulates grain width, consequently improving grain length‐to‐width ratio and appearance quality. GLW9 binds to the *EXPA6* promotor to upregulate its expression, thereby positively regulating cell expansion and grain shape. On the other hand, GLW9 directly upregulates the expression of *OsPIN1b* to reduce tiller angle. This study elucidates the mechanism by which *GLW9* coordinately regulates grain shape and tiller angle, providing theoretical reference and gene resources for the improvement of grain shape and tiller angle in rice.

## Introduction

Rice (*Oryza sativa*. L) is a staple food sustaining more than one‐third of the world's population. Rice yield is determined by multiple factors, including plant architecture, spikelet number, and grain shape. Grain shape, which is controlled by grain length (GL) and grain width (GW), is a critical factor determining rice yield and quality (Xing and Zhang, 2010). Up to now, large numbers of studies demonstrate that grain shape is regulated by multiple pathways, involving G‐protein signalling, ubiquitin‐proteasome pathways, MAPK signalling, plant hormone sensing and signalling as well as transcription factors (Fan and Li, 2019; Ren *et al*., 2023). Despite significant progress has been achieved, the genetic and molecular mechanisms underlying grain shape remain to be further elucidated in order to provide abundant gene resources and clear molecular pathways for design breeding of rice grain shape in the future.

Rice grain shape is mainly restricted by spikelet hull size, which is attributed to cell proliferation and expansion (Fan and Li, 2019; Ren *et al*., 2023). In plants, cell expansion is primarily attributed to the action of expansins, a family of proteins that regulate cell expansion by facilitating the relaxation and extension of cell walls (Cosgrove, 1999; McQueen‐Mason and Cosgrove, 1994). A total of 56 expansin genes have been predicted in the rice genome, consisting of 33 EXPA genes, 18 EXPB genes, 4 EXLA genes, and 1 EXLB gene (Cosgrove, 2015). Several expansin genes have been functionally characterized as regulators of cell expansion. The expression of *OsEXP4* is upregulated by OsNAC3, thereby promoting embryo cell elongation and seed germination (Huang *et al*., 2023). *OsEXPA8*, *OsEXPA17*, and *OsEXPB2* regulated cell expansion to control rice growth and root system architecture (Wang *et al*., 2014; Yu *et al*., 2011; Zou *et al*., 2015). *OsEXPA7* was regulated by methyl jasmonate (MeJA), brassinosteroids (BRs), and gibberellins (GAs), positively influencing cell elongation and GW (Zhang *et al*., 2024). However, the mechanisms by which expansin genes are regulated to influence grain shape largely remain unclear.

Tiller angle is a critical architectural component in rice, closely associated with shoot gravitropism, which is established as a result of asymmetric distribution of auxin that triggered by gravity perception and signal transduction. Several genes participating in auxin transport and asymmetric distribution upon gravistimulation play important roles in regulating rice shoot gravitropism and tiller angle. *LAZY1* (*LA1*) modulates stem gravitropism and tiller angle by negatively regulating polar auxin transport (Li *et al*., 2007; Yoshihara and Iino, 2007). *LAZY2* (*LA2*) and *LAZY3* (*LA3*), acting upstream of *LA1*, mediate the gravitropic response and tiller angle in rice by influencing the lateral transport of auxin (Cai *et al*., 2023; Huang *et al*., 2021). The transcription factors *WOX6* and *WOX11* serve as core regulators of rice tiller angle through affecting the asymmetric distribution of auxin (Zhang *et al*., 2018). *BTA2* influences tiller angle by controlling the content and distribution of auxin in shoot base (Li *et al*., 2024b). The PIN‐FORMED (PIN) protein family, which is functionally characterized as auxin efflux transporter, has been identified as a crucial determinant of auxin transport and distribution (Bennett, 2015; Zhang *et al*., 2020). A total of 12 *PIN* genes have been predicted in rice genome, most of which regulate rice tiller angle by affecting asymmetric auxin distribution (Chen *et al*., 2012; Wang *et al*., 2009; Xu *et al*., 2005). Nevertheless, the mechanisms by which these PIN genes are regulated to affect rice tiller angle are awaited to be investigated.

DOF (DNA binding with one finger) proteins are members of a plant‐specific transcription factor family, composed of approximately 200–400 amino acids (Riechmann *et al*., 2000). The N‐terminal of DOF proteins contains a conserved zinc finger domain that is important for DNA binding, whereas the C‐terminal harbours a transcriptional regulatory domain (Zou and Sun, 2023). The conserved core AAAG sequences or their reverse complement CTTT motifs have been consistently identified in the binding sites of DOF proteins reported previously (Yanagisawa, 2002). DOF proteins have been widely demonstrated to influence plant growth and development through the regulation of cell proliferation and expansion (Yanagisawa, 2002). DOF proteins have also been empirically demonstrated to respond to various hormonal signals, such as indole‐3‐acetic acid (IAA) (Baumann *et al*., 1999; Hu *et al*., 2022), GA (Mena *et al*., 2002; Washio, 2003), BR (Wei *et al*., 2017), abscisic acid (ABA) (Lorrai *et al*., 2018; Ramachandran *et al*., 2020; Xu *et al*., 2020) and ethylene (Qin *et al*., 2019; Wang *et al*., 2016), thereby participating in the regulatory of associated biological processes. A total of 30 *DOF* genes have been predicted in rice genome (Lijavetzky *et al*., 2003), which play pivotal roles in regulating root development (Qin *et al*., 2019; Wang *et al*., 2016), nutrition uptake (Iwamoto and Tagiri, 2016), stress response (Gandass *et al*., 2022), disease resistance (Kim *et al*., 2021; Wu *et al*., 2022b), photosynthesis (Zhang *et al*., 2015) and seed development (Kawakatsu *et al*., 2009; Wu *et al*., 2022a). However, a substantial proportion of DOF proteins in rice remains functionally uncharacterized.

In this study, we cloned a grain shape gene *Grain Length and Width on chromosome 9* (*GLW9*), which encodes OsDOF25, from a recombinant inbred line (RIL) population derived from a cross of *indica* cultivar Jin23b (J23B) and *japonica* cultivar Beilu130 (BL130) by map‐based cloning. Transgenic experiments confirmed that *GLW9* has a positive effect on GL whereas a negative effect on GW and tiller angle. Scanning electronic microscope (SEM) observation results indicated that *GLW9* regulates cell expansion to control grain shape, and *EXPA6* is a direct downstream target of GLW9 in regulating cell expansion, which was validated through dual luciferase reporter assays, ChIP‐qPCR, and EMSAs. Moreover, hormone response experiments, transgenic experiments and interaction assays demonstrated that *GLW9* negatively regulates tiller angle by upregulating the expression of a direct downstream target gene, *OsPIN1b*. Our findings provide new insights into the molecular mechanism underlying the control of grain shape and tiller angle in rice.

## Results

### Map‐based cloning of *GLW9*

**Figure 1:**
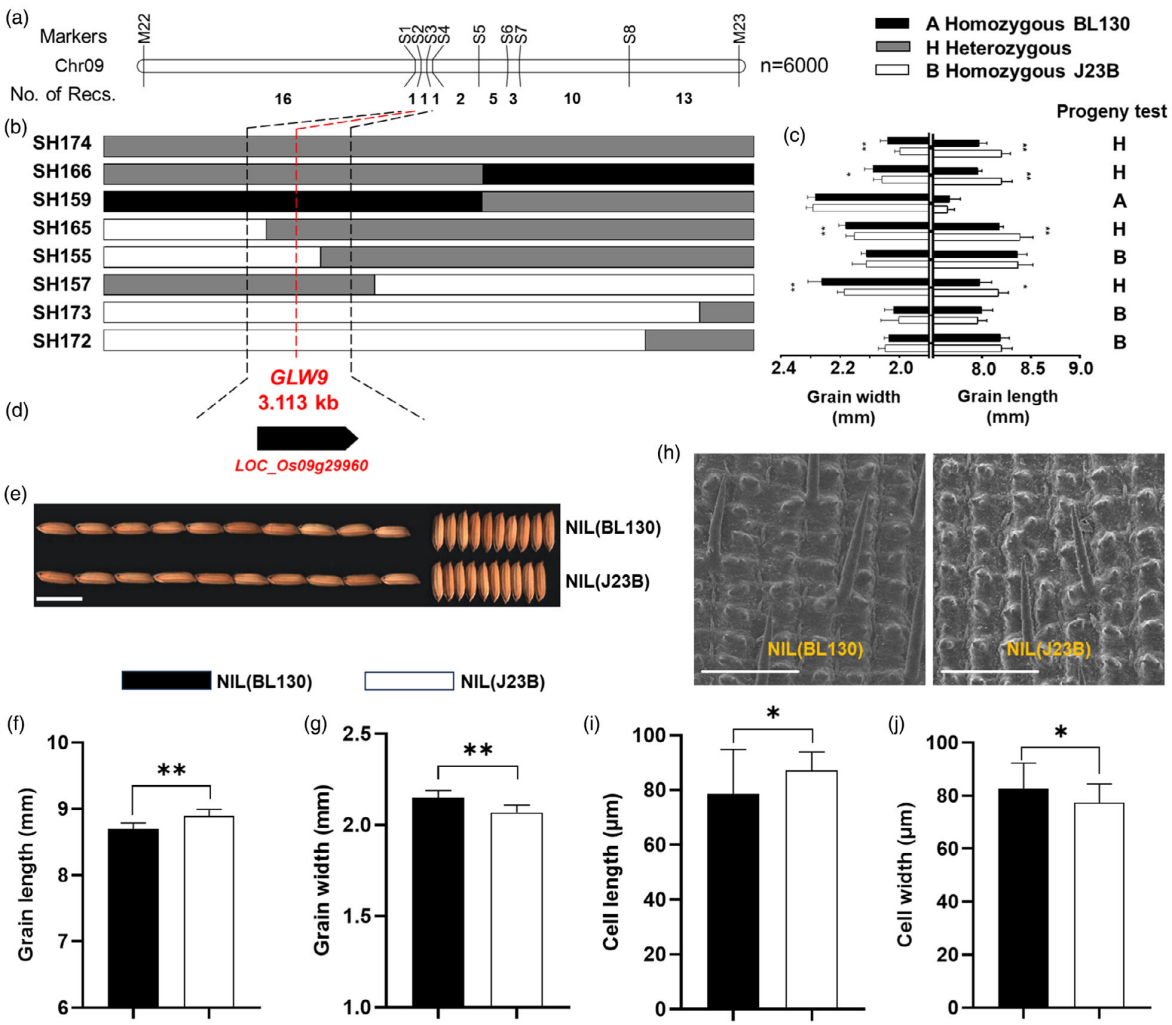
Fine mapping of *GLW9*. (a) The mapping region of *GLW9* on chromosome 9. n represents the number of plants used for recombinants screening. No. of Recs. indicates the number of recombinants between flanking markers. (b, c) Genotypes (b) and phenotypes (c) of representative recombinants. Progeny test result was defined based on the comparison of GL and GW differences between two types of homozygous genotypes of each recombinant. If the differences were significant, the *GLW9* genotype was defined as ‘H’; if the differences were not significant, the *GLW9* genotype was defined as ‘A’ or ‘B’. The red dash line indicates the co‐segregation marker S2. (d) Gene annotation information in the 3.113 kb region. (e–g) Comparison of grain shape (e), GW (f) and GL (g) between NILs. Scale bars: 10 mm. (h–j) Comparison of cell size (h), cell length (i) and cell width (j) between NILs. Scale bars: 200 μm. Significant differences were based on two‐tailed *t*‐tests. *P < 0.05 and **P < 0.01, respectively.

In a previous study, we identified a QTL for GL, namely *qGL9*, in the RIL population derived from the cross between J23B and BL130 (Yun *et al*., 2016). The *qGL9* allele from J23B increased GL and decreased GW simultaneously. To fine map *qGL9*, we developed a segregating BC_5_F_2_ population consisting of 6000 plants and identified 53 recombinants between markers M22 and M23 (Figure 1a). Progeny test of all recombinants was conducted, and both GL and GW were used to deduce the genotype of *qGL9* (Figure 1b, c). The *qGL9* genotypes of four lines, namely SH174, SH166, SH165 and SH157, were regarded as heterozygous (H), indicating that the candidate gene was located in the chromosomal region between markers S1 and S4. The genotypes of SH159, SH155, SH173 and SH172 were regarded as homozygous (A or B), suggesting that the candidate gene was located in the chromosomal region upstream of marker S3. In summary, the candidate gene for *qGL9* was located within the 3.113 kb region flanked by markers S1 and S3 and co‐segregated with marker S2 (Figure 1b). According to the annotation information of reference Nipponbare genome (Rice Genome Annotation Project, https://rice.uga.edu/), the 3.113 kb region contains the full‐length of *LOC_Os09g29960* that encodes OsDOF25, a member of rice DOF transcription factor family (Figure 1d). Hence, we designated *LOC_Os09g29960* as the candidate gene for *qGL9* and functionally named it *GLW9*.

To precisely evaluate the effect of *GLW9* on grain shape, we examined the GL and GW of NIL(BL130) and NIL(J23B), a pair of NIL for *GLW9* derived from the BC_7_F_2_ generation in the J23B background. NIL(J23B) had a higher GL value but a lower GW value compared to NIL(BL130) (Figure 1e–g). SEM observation of the lemma showed that the cell length in NIL(J23B) was significantly longer than that in NIL(BL130), whereas the cell width was narrower, with no significant difference observed in cell number (Figure 1h–j, Figure S1). Hence, these results indicated that *GLW9* modulates grain shape by affecting cell size rather than cell number.

### *GLW9* positively regulates grain length‐to‐width ratio

**Figure 2:**
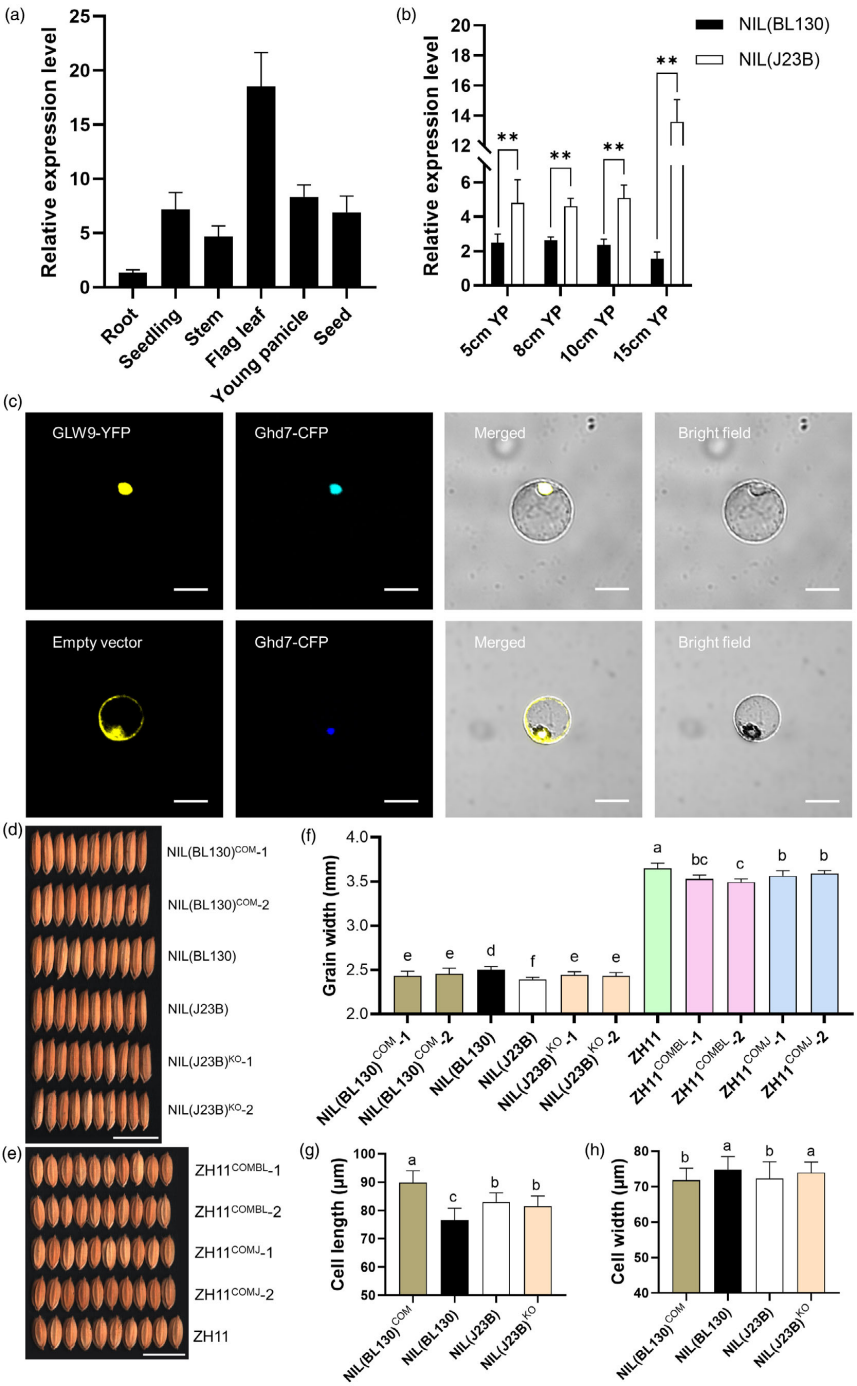
Expression patterns, subcellular localization and transgenic verification of *GLW9*. (a) Spatiotemporal expression patterns of *GLW9* in J23B plants. Root and seedling were roots and shoots of 5 cm seedlings. Stem and flag leaf were sampled at flowering stage. Seed was the caryopsis 7 days after flowering. (b) Comparison of *GLW9* expression level in young panicles between NILs. Significant differences were based on two‐tailed *t*‐tests. **P < 0.01. (c) GLW9‐YFP and Ghd7‐CFP (nuclear marker) were co‐located in the nucleus. The empty vector pM999‐YFP was used as a negative control. Scale bars: 20 μm. (d, e) Comparison of grain width (GW) among NILs and *GLW9* transgenic plants under J23B (d) and ZH11 (e) background. Scale bars: 10 mm. (f) Comparison of GW among different lines. Significance of differences was determined by Duncan's multiple range test. (g, h) Comparison of cell length (g) and cell width (h) in the lemma among different lines. Significance of differences was determined by Duncan's multiple range test.

The 3.113 kb interval encompasses the 337 bp promotor region and the full‐length coding region of *GLW9*. We conducted qRT‐PCR to examine the expression pattern of *GLW9* in different tissues. *GLW9* was expressed in all detected tissues, with the highest expression level in flag leaves, followed by young panicles, seedlings, seeds, stems and roots (Figure 2a), suggesting that *GLW9* might exert its functions in various tissues. Further examination of *GLW9* expression in young panicles of varying lengths revealed that the expression level of *GLW9* in NIL(J23B) was significantly higher than that in NIL(BL130) across the whole development stage of young panicles (Figure 2b). Consequently, *GLW9* positively regulates GL and negatively regulates GW simultaneously.

*GLW9* encodes a DOF transcription factor which might function in the nucleus. We conducted transient expression of *GLW9* in rice protoplasts to analyse its subcellular localization. GLW9‐YFP protein was co‐located with nuclear marker Ghd7‐CFP in rice protoplast (Figure 2c), indicating that GLW9 functions in the nucleus to regulate the expression of downstream genes involved in grain shape regulation.

To verify that *GLW9* is responsible for the grain shape variation, we conducted several transgenic experiments. A complementation vector CO^J23B^ (*GLW9* coding region from J23B driven by its native 2 kb promotor; Figure S2a) was transformed into NIL(BL130) plants. Compared to NIL(BL130), the T_1_ generation transgenic‐positive lines NIL(BL130)^COM^‐1 and NIL(BL130)^COM^‐2 exhibited higher *GLW9* expression levels, as well as larger GL and lower GW values (Figure 2d, f; Figures S2e and S3a, b). Two knockout (KO) plants in NIL(J23B) background were generated by CRISPR Cas9 genome editing (Figure S2c, d, g). The T_1_ generation KO lines NIL(J23B)^KO^‐1 and NIL(J23B)^KO^‐2 displayed significantly lower GL and larger GW values than NIL(J23B) (Figure 2d, f; Figure S3a, b). SEM observation of the lemma of NILs and transgenic lines confirmed that *GLW9* confers longer and narrower cells (Figure 2g, h; Figure S3c–h).

Furthermore, two complementation vectors, CO^J23B^ and CO^BL130^ (*GLW9* coding region from BL130 driven by its native 2 kb promotor; Figure S2b) were transformed into ZH11 plants, respectively. The expression levels of *GLW9* in these two transgenic positive plants were significantly higher than that in ZH11, resulting in a significant decrease in GW, while no significant difference in GL was observed (Figure 2e, f; Figure S2f; Figure S3a, i). Therefore, the above results confirmed that *GLW9* influences cell size, thereby positively regulating GL and negatively regulating GW, ultimately resulting in an increase in LWR.

### Effects of *GLW9* on agronomy traits and haplotype analysis

**Figure 3:**
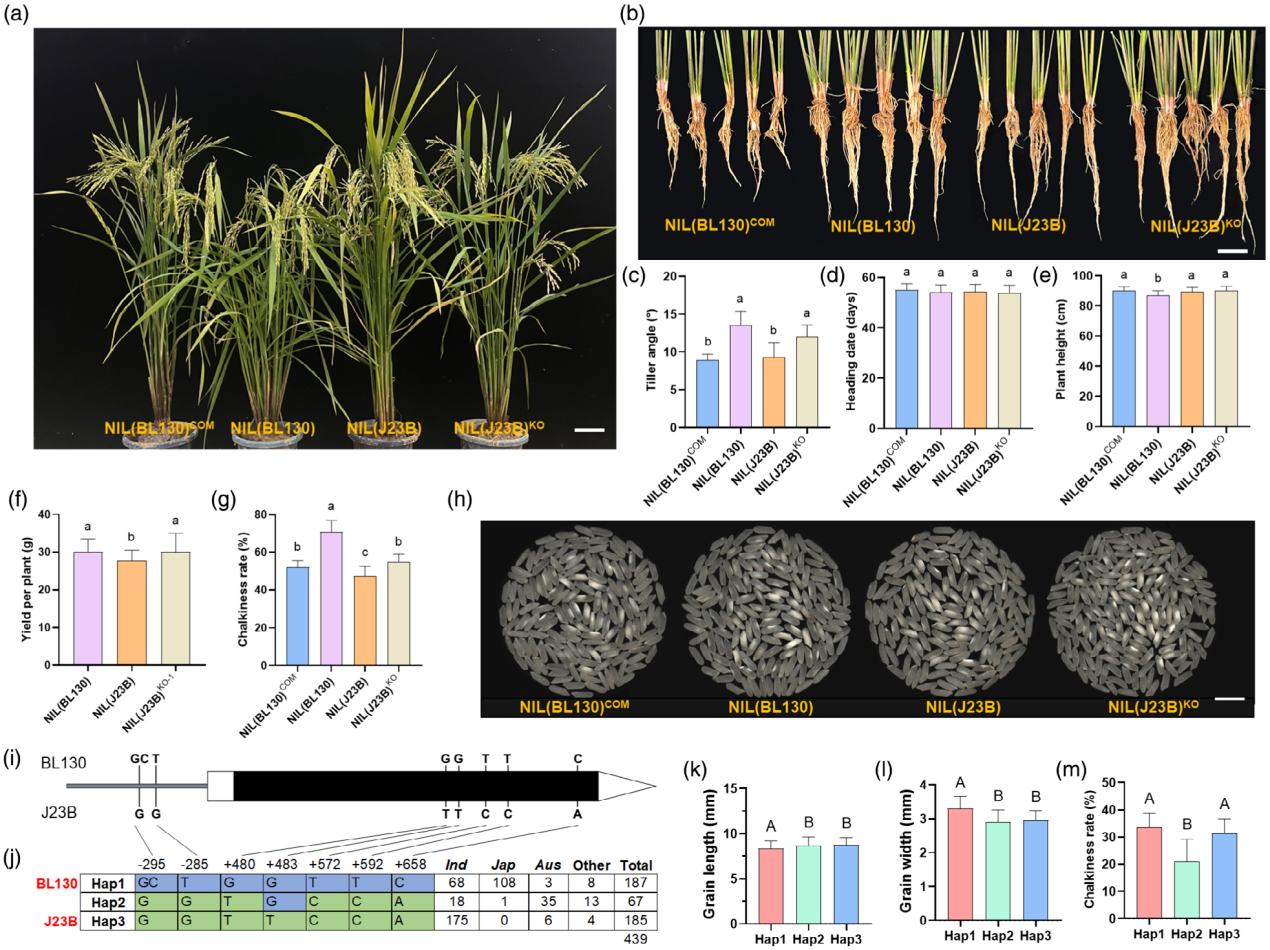
Comparison of agronomy traits among different lines and natural variation analysis of *GLW9*. (a, b) Plant architecture (a) and tiller anger (b) of NILs and *GLW9* transgenic lines. Scale bars: 10 cm. (c–g) Comparison of tiller angle (c), heading date (d), plant height (e), yield per plant (f) and chalkiness rate (g) among NILs and transgenic lines. (h) Chalkiness of NILs and transgenic lines. Scale bar: 10 mm. (i) Sequence variations of *GLW9* between J23B and BL130. (j) Haplotype analysis of *GLW9* in 439 cultivated rice accessions. (k–m) Comparison of GL (k), GW (l) and chalkiness rate (m) among different haplotypes. Significance of differences was determined by Duncan's multiple range test.

To evaluate the effect of *GLW9* on rice growth and development, we investigated the agronomic traits of NILs and transgenic lines. The tiller angles of NIL(J23B) and NIL(BL130)^COM^ were significantly lower than that of NIL(BL130), while knockout of *GLW9* significantly increased tiller angle (Figure 3a–c). We examined the main stems of NILs and transgenic lines and found that the stem nodes of the main stems of NIL(BL130) and NIL(J23B)^KO^ plants with larger tiller angles mostly showed a curved morphology (Figure S4a). We used semi‐thin sectioning technology to section the stem nodes of each line and then conducted cytological observations. It was found that there were no significant differences in the number and width of cells within the stem nodes among different lines (Figure S4b–d). Further observation and analysis revealed that there were more and larger axillary bud organs on the stem nodes of NIL(BL130) and NIL(J23B)^KO^ plants with a greater degree of curvature (Figure S4e). Therefore, more developed axillary bud organs may lead to greater morphological changes at the stem nodes, causing stem bending and an increase in tiller angle.

We then analysed the effects of *GLW9* on yield‐related traits in NILs and transgenic plants. No significant effect on heading date was observed, and significant change in plant height was detected only in NIL(BL130) (Figure 3a, d, e; Table S1). In terms of yield‐related traits, there were no significant differences in effective panicles per plant and setting rate among different lines, whereas spikelet number per panicle and filled grain number per panicle in NIL(J23B) were significantly lower than those in NIL(BL130) and knockout lines (Figure S5a–d; Table S1). Moreover, the LWR of NIL(BL130) and NIL(J23B)^KO^ lines was significantly lower than that of NIL(J23B), whereas the 1000‐grain weight did not exhibit a significant change (Figure S5e, f; Table S1). As a result, the yield per plant of NIL(J23B) showed a significant decrease compared to NIL(BL130) and NIL(J23B)^KO^ (Figure 3f). Given that the rice grain shape is closely related to grain appearance quality, we examined the chalkiness rate of NILs and transgenic lines. NIL(J23B) and NIL(BL130)^COM^, which had a higher LWR, exhibited a lower chalkiness rate compared to NIL(BL130) and NIL(J23B)^KO^ (Figure 3g, h). Therefore, *GLW9* negatively regulates the yield per plant but positively regulates the appearance quality of rice.

In the 3.113 kb candidate interval of *GLW9*, 2 and 5 sequence variations were identified in the promoter region and coding region, respectively (Figure 3i). To uncover the functional variation underlying *GLW9*, we analysed the 7 sequence variations and grain shape of 439 cultivated rice accessions. A total of three haplotypes were identified, with BL130 and J23B belonging to Hap 1 and Hap 3, respectively (Figure 3j). The *japonica* accessions primarily belong to the Hap 1 (Figure 3j), exhibiting distinct subspecies specificity. Hap 2 and Hap 3 exhibited significantly increased GL and decreased GW than Hap 1 (Figure 3k, l). However, Hap2 displayed a lower chalkiness rate than Hap1 and Hap3 (Figure 3m). Given that *japonica* accessions typically have a wider GW and higher chalkiness, introducing the Hap 2 alleles of *GLW9* into *japonica* rice may have a positive effect on improving its appearance quality by simultaneously reducing GW and chalkiness.

### Transcriptional regulation analysis of *GLW9* on expansins

**Figure 4:**
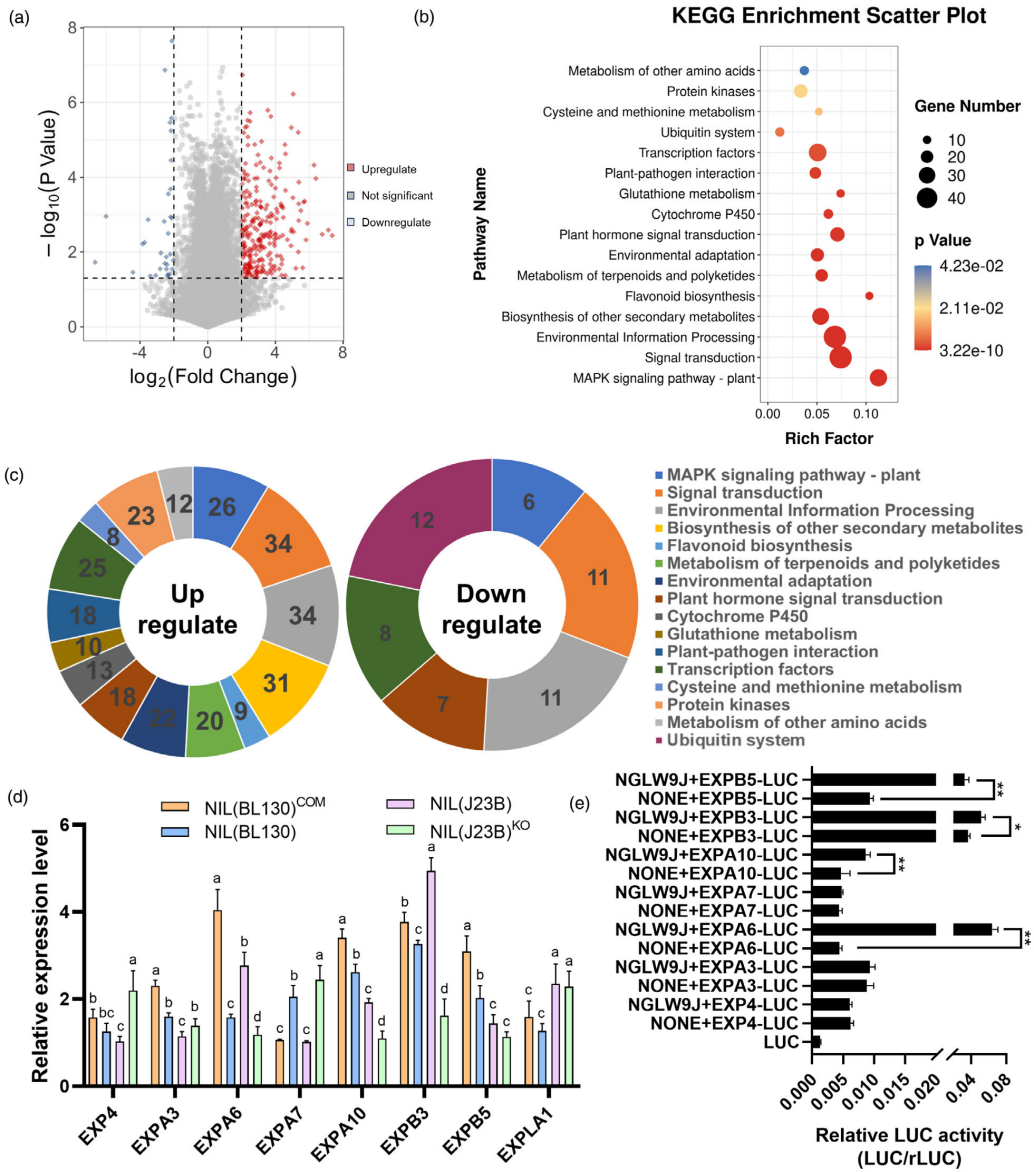
GLW9 regulates the expression of expansin genes. (a) Volcano plot of DEGs between NILs. (b, c) KEGG enrichment of differentially expresed genes. The pie charts (c) show the number of up‐regulated (left) and down‐regulated (right) genes for each KEGG pathway in NIL(BL130). (d) Differential expression analysis of expansin genes in NILs and *GLW9* transgenic lines. Significance of differences was determined by Duncan's multiple range test. (e) Regulation analysis of GLW9 on the promotor of expansin genes. Significant differences were based on two‐tailed *t*‐tests, *P < 0.05 and **P < 0.01, respectively.

The *GLW9*‐encoded OsDOF25 has been shown to have transcriptional activation activity on downstream genes (Kushwaha *et al*., 2011; Zhang *et al*., 2015). To elucidate the mechanism by which *GLW9* influences grain shape, we conducted RNA‐seq analysis on 5 cm young panicles of NILs. The results showed that most differentially expressed genes (DEG) were upregulated in NIL(BL130) (Figure 4a). KEGG enrichment analysis of DEGs revealed that *GLW9* influenced multiple grain shape related pathways, including MAPK signalling pathway, signal transduction, plant hormone signal transduction, transcription factors and protein kinases (Figure 4b). Further analysis demonstrated that more DEGs involved in grain shape‐related pathways were upregulated in NIL(BL130), whereas all DEGs related to ubiquitin system were upregulated in NIL(J23B) (Figure 4c). These results suggested that *GLW9* might regulate grain shape by affecting the expression of genes involved in multiple pathways.

Expansins are a family of proteins that regulate cell expansion in the cell wall (Cosgrove, 1999; McQueen‐Mason and Cosgrove, 1994). Due to the significant influence of *GLW9* on cell size in regulating grain shape, we analysed the expression of expansin genes in the RNA‐seq data of NILs and selected 8 expansin genes with significant expression differences for further qRT‐PCR analysis. The results showed that the expression of the 8 expansin genes was significantly changed among NILs and transgenic plants (Figure 4d). We further used a dual luciferase reporter system to analyse the transcriptional regulation of *GLW9* on the 8 expansin genes in rice protoplast (Figure S6). The results demonstrated that GLW9 could activate the expression of *EXPA6*, *EXPA10*, *EXPB3* and *EXPB5* (Figure 4e). Among them, only the expression patterns of *EXPA6* and *EXPB3* are consistent with those observed in the young panicles. Notably, the expression of *EXPA6* in protoplasts can be strongly activated by GLW9. Thus, we propose that *EXPA6* could be a direct downstream target gene of GLW9.

### GLW9 directly upregulates the expression of *EXPA6* to affect grain shape

**Figure 5:**
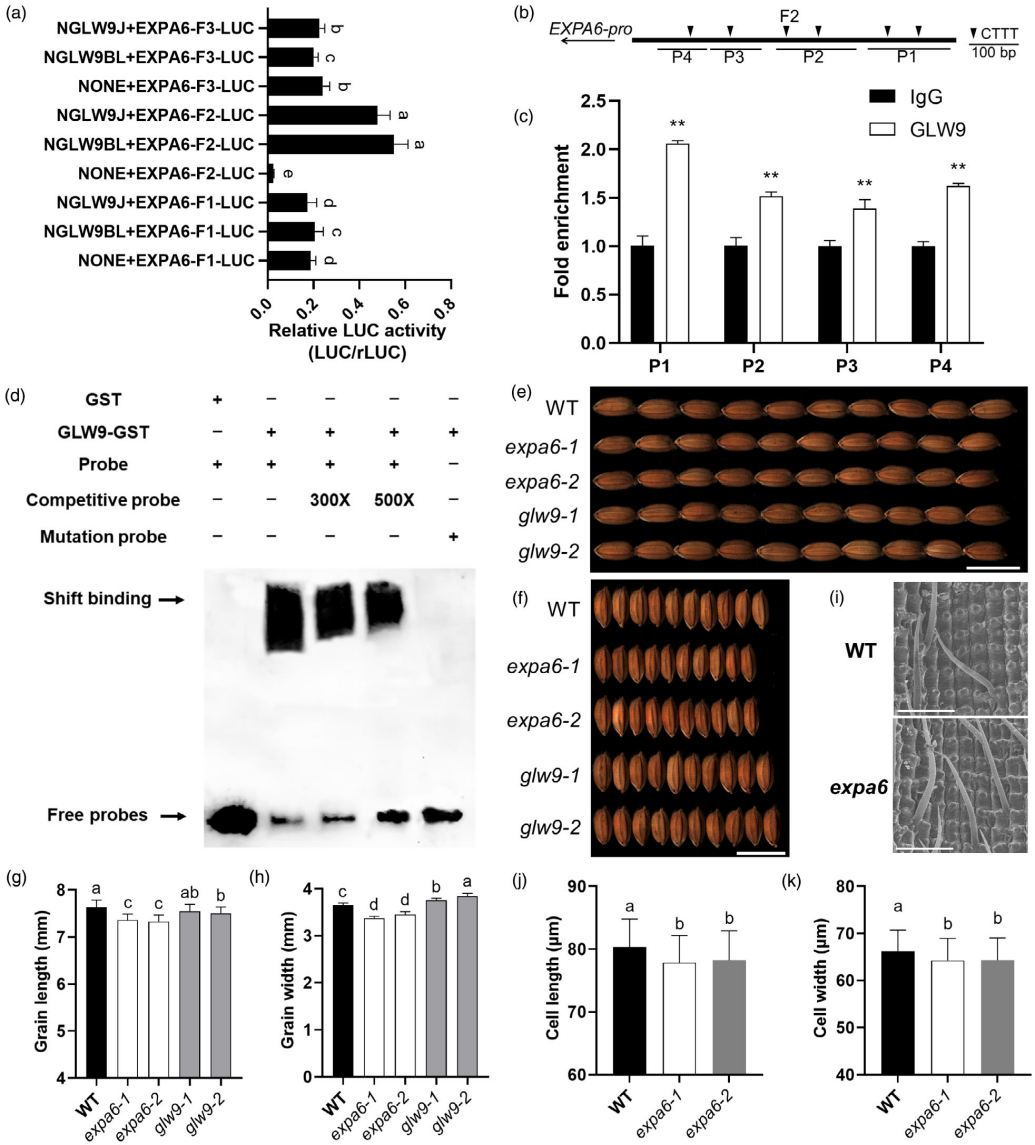
GLW9 upregulates *EXPA6* expression to positively affect grain shape. (a) Regulation analysis of GLW9 on different *EXPA6* promotor fragments. Significance of differences was determined by Duncan's multiple range test. (b) Schematic representation of the putative locations of GLW9‐binding sites in the promotor of *EXPA6*. The arrow indicates the direction of transcription. (c) ChIP‐PCR assays showing the GLW9‐binding regions in the *EXPA6* promoter *in vivo*. Significant differences were based on two‐tailed *t*‐tests, **P < 0.01. (d) EMSAs of GLW9 binding to the cis‐elements of *EXPA6*. Lane 1, no GLW9 fusion protein; lanes 2–5, fusion protein with Probe 1, competitive probe, and Probe 2 (from AAAG to CCCT), as indicated. (e–h) Comparison of grain length (e, g) and grain width (f, h) among wild‐type (WT), *glw9* and *expa6* lines. Scale bars: 10 mm. (i–k) Comparison of cell size (i), cell length (j) and cell width (k) among WT and *expa6* lines. Scale bars: 200 μm. Significance of differences was determined by Duncan's multiple range test.

The AAAG motif (or reverse complement CTTT motif) was previously reported to be the core binding element of DOF family transcription factors (Yanagisawa, 2002), and 15 CTTT motifs were identified in the 2 kb promotor region of *EXPA6* (Figure S7a). To investigate the binding site of GLW9, the *EXPA6* promotor was divided into three fragments, which were separately ligated to the dual luciferase reporter plasmid 190LUC (Figure S7a, b), and then were subjected to analysis. The results showed that GLW9 only significantly activated the transcription of reporter carrying fragment 2 (Figure 5a). To further ascertain the binding site of GLW9 on the *EXPA6* promotor *in vivo*, we transformed the GLW9‐YFP fusion plasmid to rice protoplasts and then performed chromatin immunoprecipitation followed by quantitative PCR (ChIP‐qPCR) assays using GFP antibody. The fragment 2 of *EXPA6* promotor was divided into four short regions (P1–P4) covering 6 CTTT motifs, and significant enrichments were found in all four regions (Figure 5b, c). In addition, the binding of GLW9 to the CTTT motif in *EXPA6* promotor was confirmed *in vitro* with electrophoretic mobility shift assays (EMSAs) (Figure 5d). These results demonstrated that GLW9 specifically binds to the AAAG motif within fragment 2 of *EXPA6* promotor.

To examine the effect of *EXPA6* on grain shape, two *expa6* knockout mutants were generated in ZH11 background by CRISPR Cas9 genome editing (Figure S8a), and both displayed significantly reduced GL and GW values relative to WT plants (Figure 5e–h). SEM observation of lemma of *expa6* and wild‐type (WT) plants revealed that *EXPA6* positively regulated cell length and cell width but exhibited no effect on cell number (Figure 5i–k; Figure S9). We also generated two *glw9* knockout mutants in the ZH11 background (Figure S8b). Compared with WT, the GL of the *glw9‐2* plants decreased significantly, whereas the GW of *glw9‐1/2* increased significantly (Figure 5e–h). In summary, these results reveal that *GLW9* directly upregulates the expression of *EXPA6* to affect cell shape, which ultimately increases grain length. However, how *GLW9* negatively regulates grain width remains unknown.

### *GLW9* positively regulates auxin‐mediated shoot gravitropism

**Figure 6:**
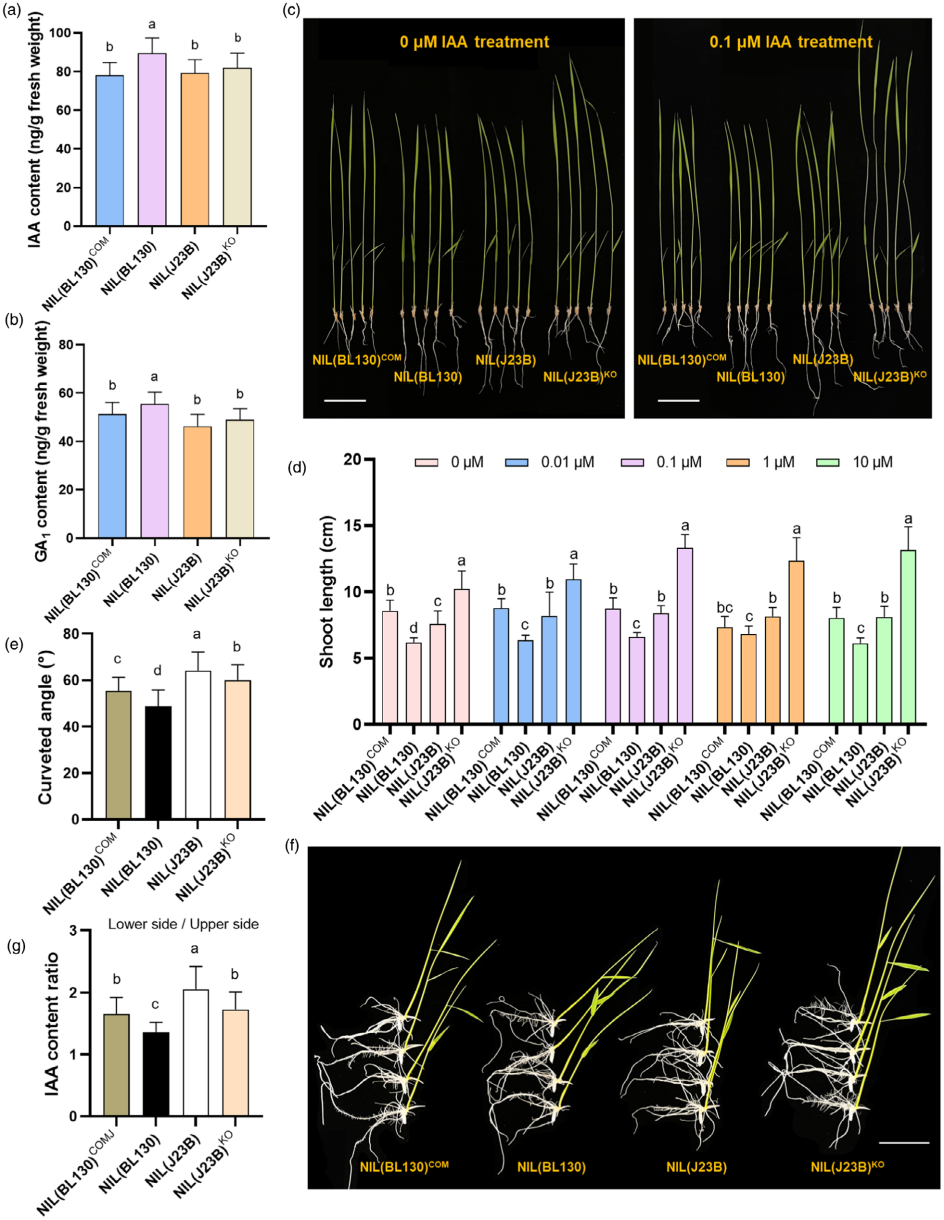
*GLW9* negatively regulates IAA response. (a, b) Comparison of IAA (a) and GA_1_ (b) content in basal node among NILs and *GLW9* transgenic lines. (c, d) Shoot length of NILs and *GLW9* transgenic seedlings under different concentrations of IAA treatment. Scale bars: 5 cm. (e, f) Shoot gravitropic response of NILs and *GLW9* transgenic lines. Scale bar: 5 cm. (g) Comparison of lower side/upper side IAA content ratio in 7‐day‐old seedlings among NILs and *GLW9* transgenic plants after 24 h for gravitropic stimulation. Significance of differences was determined by Duncan's multiple range test.

Tiller angle is closely related to plant hormones. As *GLW9* exerted a negative effect on tiller angle (Figure 3a–c), we measured the content of several plant hormones, namely CTK, BR, IAA, GA_1_, GA_2_ and GA_3_ in the basal node of NILs. Significant differences were found in the content of IAA, GA_1_ and GA_3_ (Figure S10). We subsequently conducted qRT‐PCR to examine the expression of genes related to GA and IAA signal conduction in the basal nodes and found that the majority of related genes were up‐regulated in NIL(BL130) compared to NIL(J23B) (Figure S11). Moreover, the contents of IAA and GA_1_ in basal nodes of NIL(J23B) and NIL(BL130)^COM^ decreased by 11.4% and 12.6%, respectively, compared to NIL(BL130), whereas KO of *GLW9* did not result in significant changes (Figure 6a, b), which might be attributed to the compensatory effect of homologous genes, or the reduced expression of *GLW9* may play a vital role in the regulation of IAA and GA_1_ content. To further explore the relationship between *GLW9* and IAA as well as GA, we treated the seedling of NILs and transgenic lines with various concentrations of exogenous IAA and GA. With the increase in IAA concentration, only the shoot length of NIL(J23B)^KO^ exhibited a significant increase, and the root growth was inhibited when the exogenous IAA concentration exceeded 0.1 μM (Figure 6c, d; Figure S12a). Moreover, an increase in GA concentration led to higher shoot length in all lines, but the differences among them gradually decreased (Figure S12b). These results suggested that *GLW9* might negatively regulate the response to IAA, whereas *GLW9* could affect the synthesis and response to GA.

The gravitropic response, which was reported to strongly influence the tiller angle of rice, is closely associated with the asymmetric distribution of auxins (Ding *et al*., 2021; Li *et al*., 2020, 2021, 2024b; Ottenschläger *et al*., 2003). To further explore the relationship between *GLW9* and IAA signal conduction, we examined the gravity‐induced shoot curvature of NILs and transgenic plants after gravistimulation at 90° to the vertical for 24 h. The average shoot curveted angle of NIL(J23B), NIL(BL130), NIL(BL130)^COM^ and NIL(J23B)^KO^ plants were 64°, 49°, 55° and 60°, respectively (Figure 6e, f), indicating that *GLW9* exerts a positive effect on it. We also examined the IAA content in lower side and upper side of seedling of NILs and transgenic lines after gravistimulation at 90° to the vertical for 24 h. The lower side/upper side IAA content ratio showed significant increase in NIL(BL130)^COM^ compared to NIL(BL130), whereas significant decrease in NIL(J23B)^KO^ compared to NIL(J23B) (Figure 6g). Taken together, the above results suggested that *GLW9* positively regulates the asymmetric distribution of IAA to enhance shoot gravitropism.

### GLW9 directly upregulates the expression of *OsPIN1b* to reduce tiller angle

**Figure 7:**
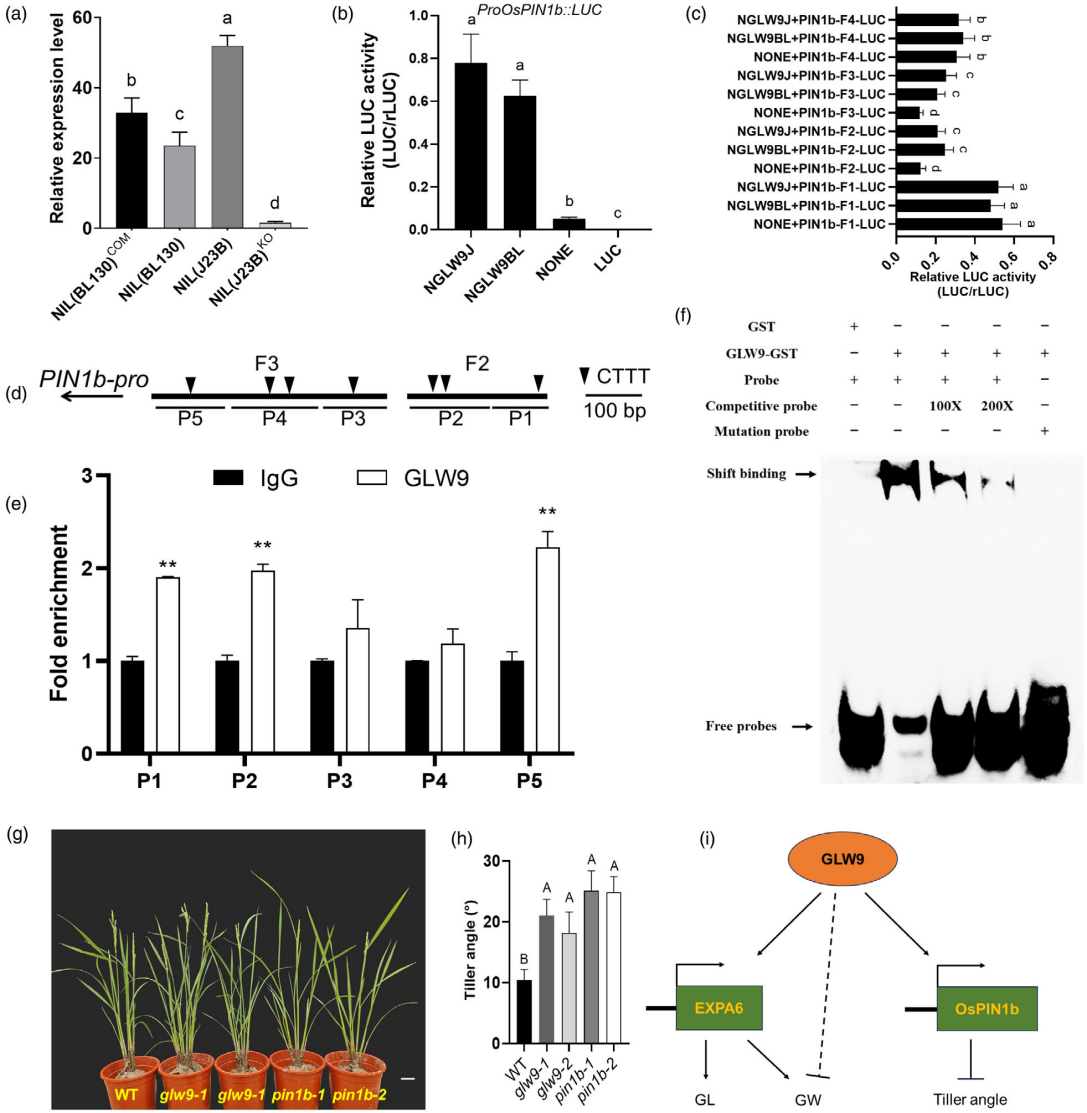
GLW9 upregulates *OsPIN1b* expression to negatively regulate tiller angle and a proposed regulation model of *GLW9* on grain shape and tiller angle. (a) Expression level analysis of *OsPIN1b* in NILs and *GLW9* transgenic lines. Significance of differences was determined by Duncan's multiple range test. (b, c) Regulation of GLW9 on *OsPIN1b* promotor. Significance of differences was determined by Duncan's multiple range test. (d) Schematic representation of the putative locations of GLW9‐binding sites in the promoter of *OsPIN1b*. The arrow represents the direction of transcription. (e) ChIP‐PCR assays showing the GLW9‐binding regions in the *OsPIN1b* promoter. Significant differences were based on two‐tailed *t*‐tests, **P < 0.01. (f) EMSAs of GLW9 binding to the *cis*‐elements of *OsPIN1b*. Lane 1, no GLW9 fusion protein; lanes 2–4, fusion protein with Probe 3, competitive probe and Probe 4 (from AAAGAAAG to CCCTCCCT), as indicated. (g, h) Tiller angle of different lines. Significance of differences was determined by Duncan's multiple range test. Scale bar: 10 cm. (i) A proposed regulatory model of GLW9 on grain shape and tiller angle. GLW9 upregulates the expression of *EXPA6* and *OsPIN1b*, positively regulating GL and negatively regulating tiller angle, respectively. However, *GLW9* negatively regulate GW through unknown mechanisms.

The asymmetrical distribution of auxin influx and efflux carriers within the plasma membrane establishes the auxin gradient and facilitates its transport, ultimately influencing the curvature of plant organs. Previous studies have suggested that PIN genes regulate rice tiller angle by affecting asymmetric auxin distribution (Chen *et al*., 2012; Wang *et al*., 2009; Xu *et al*., 2005), and *OsPIN1b* is involved in the regulation of auxin transport, negatively regulating axillary bud formation and tiller number (Li *et al*., 2022; Sun *et al*., 2018). We discovered that *GLW9* positively regulated the expression of *OsPIN1b* in the basal nodes (Figure 7a; Figure S11), implying that GLW9 may reduce the tiller angle by negatively regulating axillary bud formation and growth (Figure S4). Thus, *OsPIN1b* might be a downstream target of *GLW9* for regulating tiller angle.

To detect the transcriptional activation effect of GLW9 on the *OsPIN1b* promotor, we conducted the dual‐luciferase reporter assays in rice protoplast. The results showed that GLW9 activated the expression of the reporter carrying the *OsPIN1b* promotor, and there was no significant difference in the activation effect on the downstream *OsPIN1b* promoter between the two parental types of GLW9 (Figure 7b). A total of 11 AAAG (or reversed CTTT) motifs were identified within the 2 kb promotor of *OsPIN1b* (Figure S13a). To analyse the binding site of GLW9 on the *OsPIN1b* promotor, it was divided into four fragments, which were ligated to the dual luciferase reporter plasmid 190LUC, respectively (Figure S13a). The results suggested that fragment 2 and fragment 3 of *OsPIN1b* promotor could be the binding targets of GLW9 (Figure 7c). Subsequently, based on the position of AAAG motifs, the fragment 2 and fragment 3 of *OsPIN1b* promotor were divided into five regions (P1–P5), which were used for ChIP‐qPCR analysis *in vivo* (Figure 7d). Significant enrichments were found in the P1, P2 and P5 region of *OsPIN1b* promotor (Figure 7e). In addition, the binding of GLW9 to the promotor of *OsPIN1b* was confirmed *in vitro* using EMSAs (Figure 7f). Consequently, the above results demonstrated that GLW9 directly binds to the promotor of *OsPIN1b* and upregulates its expression.

To investigate the effect of *GLW9* regulation on *OsPIN1b* in relation to tiller angle, we examined the tiller angle in the background of ZH11 for the *glw9* and *pin1b* knockout lines and found that KO of *GLW9* and *OsPIN1b* significantly increased tiller angle (Figure 7g, h), indicating that *OsPIN1b* is genetically located downstream of GLW9. *OsPIN1b* has been confirmed to regulate IAA transport, thereby negatively regulating axillary bud formation (Li *et al*., 2022). Therefore, GLW9 inhibits the formation and growth of axillary buds by positively regulating the expression of *OsPIN1b*, ultimately exerting a negative regulatory effect on tiller angle.

## Discussion

The transcription factor regulatory pathway is a crucial determinant of agronomic traits in rice, and transcription factors can concurrently regulate multiple traits according to the variations in expression patterns or downstream target genes. For instance, OsSPL14, encoding a plant‐specific transcription factor that also knows as *IDEAL PLANT ARCHITECTURE1* (*IPA1*) or *WEALTHY FARMER'S PANICLE* (*WFP*), can directly and positively regulate *DEP1* to influence plant height and panicle length, and activate the expression of *WRKY45* to enhance disease resistance in rice (Lu *et al*., 2013; Wang *et al*., 2018). In this study, we map‐based cloned *GLW9*, which encodes OsDOF25, a member of DOF family transcription factors (Figure 1d). DOF transcription factors have been reported to influence multiple traits in rice, such as heading date (Li *et al*., 2009), photoperiod regulation (Wu *et al*., 2017a), ammonium uptake and transport (Wu *et al*., 2017b), disease resistance (Wu *et al*., 2018), leaf senescence (Shim *et al*., 2019) and root development (Qin *et al*., 2019). Previous studies have shown that OsDOF25 can activate the expression of the C4 photosynthesis gene *OsC4PPDK* (Zhang *et al*., 2015). We revealed that GLW9 or OsDOF25 directly up‐regulates the expression of *EXPA6* to enlarge grain shape (Figure 5) and simultaneously directly up‐regulates the expression of *OsPIN1b* to reduce tiller angle (Figure 7). Thus, *GLW9*/*OsDOF25* is a pleiotropic gene that regulates rice grain shape and tiller angle. The findings of the present study could expand our understanding of the functions of OsDOF25 and other DOF transcription factors in rice.

The cytology basis of rice grain shape variation lies in changes in cell size or cell number. Cell size or cell expansion is directly regulated by expansins, which have been reported to have a positive impact on grain shape (Fan and Li, 2019; Ren *et al*., 2023). For instance, *OsEXPB2* and *OsEXPA7* have been shown to positively regulate cell expansion and grain shape (Zhang *et al*., 2024; Zou *et al*., 2015), whereas the degradation of OsEXPLA1 leads to a reduction in grain size (Choi *et al*., 2018). In this study, we found that *GLW9* increases cell length and decreases cell width but has no effect on cell number (Figure 2g–h; Figure S1). We further demonstrated that GLW9 directly binds to the promotor of *EXPA6* to upregulate its expression, thereby positively regulating cell expansion and grain shape (Figure 5e–k). It should be noted that the effect of *EXPA6* on GW is not consistent with that of *GLW9*, implying that other unknown genes are involved in *GLW9*‐mediated regulation of GW. Notably, we found that *GLW9* possesses transcriptional activation activity (Figure 4e; Figure 7b), which is consistent with previous studies (Kushwaha *et al*., 2011; Zhang *et al*., 2015). However, RNA‐seq analysis demonstrated that the majority of grain shape‐related DEGs were up‐regulated in young panicles of NIL(BL130) (Figure 4c), suggesting that these genes may not be directly regulated by *GLW9* and that this upregulation could potentially contribute to the increased GW in NIL(BL130). In short, this study identified *EXPA6* as a downstream target involved in *GLW9*‐mediated regulation of GL; however, the mechanism by which *GLW9* negatively regulates GW remains unknown.

Tiller angle, which is closely associated with rice yield, is strongly influenced by the gravitropism of the shoot. Previous studies have demonstrated that the asymmetric distribution of auxin is a key determinant of gravitropism (Cai *et al*., 2023; Huang *et al*., 2021; Li *et al*., 2007, 2024b; Yoshihara and Iino, 2007). In the basal node of NIL(BL130), the auxin content was significantly higher than that in the other three lines (Figure 6a), but the lower side/upper side IAA content ratio was the lowest among seedling of lines (Figure 6e–g), suggesting that *GLW9* positively regulates the asymmetric distribution of auxin in the basal node, ultimately leading to a decrease in gravitropism. Auxin polar transport in plants is mediated by auxin transporter proteins, and membrane‐localized PIN proteins are believed to define the direction and rate of auxin transport. In rice, PIN proteins have been shown to influence plant architecture by regulating auxin polar transport and asymmetric distribution. For example, *pin1a/pin1b* double mutant exhibited a significantly increased panicle branch angle compared to WT (Yong *et al*., 2019). Suppression of *OsPIN5b* led to a substantial increase in tiller angle (Lu *et al*., 2015). Overexpression of *OsPIN2* results in reduced plant height and increased tiller angle (Chen *et al*., 2012). Therefore, different PIN proteins may play distinct roles in regulating plant architecture. In this study, we observed a significant decrease in the expression of *OsPIN1b* and *OsPIN3t* in the NIL(BL130) with a larger tiller angle, while the expression of *OsPIN1c* and *OsPIN1d* significantly increased (Figure S10), suggesting that PIN family proteins may play distinct roles in regulating IAA distribution. *GLW9* has been found to potentially reduce tiller angle by negatively regulating axillary bud formation and growth (Figure S4e), while *OsPIN1b* has been confirmed to negatively regulate axillary bud formation by influencing auxin transport (Li *et al*., 2022). Moreover, a series of experiments confirmed that GLW9 directly binds to the promoter of *OsPIN1b* and activates its expression (Figure 7c–f). Transgenic experiments have demonstrated that the knockout of *GLW9* or *OsPIN1b* increases the tiller angle (Figure 7g, h). All results above support that *OsPIN1b* is a downstream target gene of GLW9 in regulating tiller angle.

Grain shape and tiller angle are critical factors influencing rice yield. In this study, we map‐based cloned *GLW9*, a new regulator that positively regulates grain LWR and negatively regulates tiller angle in rice. *GLW9* encodes a DOF family transcription factor OsDOF25, which increases cell length and grain length by directly upregulating the expression of the expansin gene *EXPA6*. Additionally, GLW9 directly upregulates the expression of the auxin efflux gene *OsPIN1b*, potentially modulating the distribution of auxin to negatively regulate rice tiller angle (Figure 7i). However, there are still unresolved questions that require further investigation. For example, the effects of *EXPA6* on GL and GW differed from those of *GLW9*suggesting that other unknown genes are involved in *GLW9*‐mediated regulation of GW. Nevertheless, this study provides new insights and genetic resources for improving rice grain shape and tiller angle.

## Materials and methods

### Plant materials and growth conditions

Rice populations were planted under natural field conditions in the experimental stations at Huazhong Agricultural University at Wuhan (N 30.49, E 114.36) in summer and at Lingshui (N 18.51, E 110.04) in winter. Twelve 30‐day‐old seedlings of each line were transplanted in the field with 16.5 cm spacing in single‐row plots; rows were 26.4 cm apart. Field management followed local practices. Ten plants from the middle of each row were harvested individually for trait measurement.

A population consisting of 6000 BC_5_F_2_ plants was grown in summer 2019 for recombinant screening. Progenies of 53 recombinants were grown in winter 2019. During 2021 and 2022 summer, BC_7_F_4_ generation NILs and T_1_ generation transgenic lines were grown at Wuhan to validate the impact of *GLW9* on grain shape. The NILs, T_2_ generation *GLW9* transgenic lines, and T_1_ generation transgenic lines of *EXPA6* and *OsPIN1b* were planted at Wuhan in 2024 for evaluating grain shape and tiller angle.

### Fine mapping of *qGLW9*

To fine map *qGLW9*, 8 newly developed markers in the interval flanked by M22 and M23 were used to conduct genotyping of the recombinants selected from a BC_5_F_2_ population of 6000 plants.

Markers for QTL mapping were designed based on sequence differences between the parents. The parental sequences within the 3.113 kb interval of *GLW9* were confirmed using Sanger sequencing technology.

Due to the relatively minor impact of *GLW9* on GL and GW, we developed a large progeny population for the fine mapping of *GLW9*. Specifically, a segregating progeny population consisting of 96 plants was cultivated for each recombinant line. In each population, 20 homozygous BL130 genotype individuals and 20 homozygous J23B genotype individuals were selected, respectively, to investigate grain shape. Seeds from three primary panicles of each individual were collected, and GL and GW were measured using high‐throughput rice phenotyping facilities (HRPF) according to the previously reported method (Yang *et al*., 2014).

### RNA extraction and qRT‐PCR

Total RNA was extracted from plant tissues using Invitrogen RNA Extraction Kit 15 596‐026 (TRIzol, USA). First‐strand cDNA synthesis was performed in a 20 mL reaction mixture containing 2 mg of RNA and 200 U of M‐MLV reverse transcriptase (Invitrogen C28025‐014, USA). qRT‐PCR was conducted on a QuantStudio 6 Flex instrument using the SYBR Green PCR reagent, following the manufacturer's instructions. Data for each sample were based on 3–5 biological replicates from different plants, with three technical replicates for each biological replicate. The rice *ACTIN1* gene was used as an internal reference for normalizing gene expression.

### Generation of transgenic rice plants

To confirm the function of *GLW9*, we obtained the 2 kb promoter fragment and a full‐length coding fragment of the *GLW9* ^*BL130*^ and *GLW9* ^*J23B*^ by PCR, respectively. The two fragments were then inserted into the plant binary vector pCAMBIA1301 using a one‐step ligation method, and the positive complementary vectors were named CO^BL130^ and CO^J23B^.

To obtain *GLW9* KO plants, the 20 bp target sequence in the exon of *GLW9* was inserted into the intermediate vector pER8‐Cas9‐U6 and cloned into pCXUN‐Cas9 according to a previously described method (Gao and Zhao, 2014). Knockout vectors for *EXPA6* and *OsPIN1* were generated using the same method.

The sequences of all vectors were confirmed by PCR and Sanger sequencing technology. These constructed vectors were introduced into *Agrobacterium tumefaciens* strain EHA105 and transformed into the relevant materials by Agrobacterium‐mediated transformation (Toki, 1997).

### Subcellular localization

The coding sequence amplified from *GLW9* cDNA was inserted into vector pM999‐YFP to generate GLW9‐YFP fusion plasmids. GLW9‐YFP and the nuclear localization marker Ghd7‐CFP were co‐transformed into rice protoplasts. Fluorescence protein imaging was performed after cultivation for 12 h of darkness using a confocal laser scanning microscopy (Leica TCSSP2, Germany). The fluorescence experiments were repeated at least three times.

### Scanning electron microscopy

Emerged husks before flowering were sampled for SEM assay. Lemmas of husks were coated with gold under vacuum conditions and observed by scanning electron microscopy (JEOL JSM‐6390LV, Japan). The cell number of lemma was counted at 32x magnification in at least six biological replicates. Cell length and width analysis was taken at 60x magnification in at least 10 biological replicates, and then the data was processed by ImageJ software. All procedures were conducted according to the manufacturer's instructions.

### Semi‐thin section

Appropriate stem nodes were collected and fixed with 50% FAA fixative (50 mL of absolute ethanol, 10 mL of 37% formaldehyde solution, 5 mL of glacial acetic acid and double‐distilled water added to a total volume of 100 mL). Semi‐thin embedding was performed using Herau Kulzer Technovit 7100 resin. The specific operational procedures refer to the method described previously (Li *et al*., 2024a).

### Transcriptome analysis

The 5 cm young panicles of NILs were collected and used for RNA‐seq analysis. Total RNA was extracted using Invitrogen RNA Extraction Kit 15 596–026 (TRIzol, USA). mRNA sequencing library construction, sequencing and differential analysis were performed by Novogene (Tianjin, China). Three biological replicates were conducted for each line.

### Haplotype analysis

Haplotypes for the 3.113 kb region of *GLW9* of 439 cultivated rice accessions were identified based on sequence variations (frequency > 0.05) in BL130 and J23B. The sequence variation data and subspecies information of *GLW9*, as well as GL and GW values of 439 accessions were obtained from RiceVarMap (http://ricevarmap.ncpgr.cn/). The chalkiness rate data of 533 accessions were derived from a previous description (Shi *et al*., 2024).

### Luciferase‐based transient transcriptional activity assay

To verify the regulation effect of *GLW9* on expansin genes, the 2 kb promotor of each expansin gene was amplified from J23B and inserted into the reporter plasmid 190LUC. Full‐length of *GLW9* from J23B was inserted into the None vector as the effector NGLW9J.

To verify the effect of two parental types of GLW9 on promotor of *EXPA6* and *OsPIN1b*, the 2 kb promotor of *EXPA6* and *OsPIN1b* from J23B was divided into three and four fragments by PCR amplification, respectively. All promotor fragments were inserted into the reporter plasmid 190LUC. Full‐length of *GLW9* from J23B and BL130 was inserted into the None vector as the effector NGLW9J and NGLW9BL, respectively.

The 35S‐driven Renilla LUC gene (*rLUC*) was used as an internal control. For each co‐transfection assay, 2 μg of reporter plasmid, 2 μg of effector plasmid and 500 ng of internal control plasmid were used. Different combinations of vectors were co‐transfected into rice protoplasts by PEG‐mediated transformation (Zhang *et al*., 2011). After incubation for 16–18 h in the dark at 28°C, relative LUC activity was measured using the Dual‐LUC Reporter Assay System (Hao *et al*., 2010). Each experiment was conducted at least six biological replicates.

### ChIP‐qPCR

For the chromatin immunoprecipitation (ChIP) assay, the 35S:GLW9‐YFP and 35S:YFP plasmids were transfected into rice protoplasts as previously described (Habiba *et al*., 2021). The protoplasts were then subjected to crosslinking for 20 min with 1% formaldehyde under vacuum. The chromatin complexes were isolated and sonicated as previously described (Saleh *et al*., 2008). ChIP products were analysed by quantitative real‐time PCR, and enrichments of the selected promoter regions of candidate genes were calculated as the ratio between the IP group and the negative control group, which were quantified by qPCR using designed region‐specific primers. Rice *Ubiquitin* was used as a reference gene.

### EMSA

The coding region of *GLW9* was amplified and cloned into pGEX‐6P plasmid for fusion with GST tag as GLW9‐GST fusion protein. The fusion protein was expressed in Escherichia coli (BL21) and purified *in vitro* using a GST Fusion Protein Purification kit (Sangon Biotech, Shanghai, China). For EMSA, we synthesized two 30‐bp DNA probes, Probe 1 and Probe 2 (mutant, from AAAG to CCCT), based on the P2 region of fragment 2 on *EXPA6* promoter sequence of J23B and labelled them at the 5′‐ends with biotin, and an unlabeled 30‐bp probe 1 for competition assays. We also synthesized another two 30‐bp DNA probes, Probe 3 and Probe 4 (mutant, from AAAGAAAG to CCCTCCCT), based on the P2 region of fragment 2 on *OsPIN1b* promoter sequence of J23B and labelled them at the 5′‐ends with biotin, and an unlabeled 30‐bp probe 3 for competition assays. Gel‐shift assays were performed according to the protocol supplied by the manufacturer (LIKEN Biotech, Sanya, China).

### Analysis of plant hormone content

The contents of IAA, CTK, BR, GA_1_, GA_3_ and GA_4_ in the basal nodes of the main stem were detected, with at least eight biological replicates conducted. The basal nodes were collected at the flowering period, and the hormone content was analysed using corresponding ELISA kit (Jingmei Biotech. Nanjing, China). Each material was subjected to at least six replicates. All procedures were conducted in accordance with the instruction's manual.

### Hormone treatments

To determine the response of *GLW9* to IAA and GA, 5‐day‐old seedlings of NILs and GLW9 transgenic lines were treated with concentrations of 0 μM, 0.01 μM, 0.1 μM, 1 μM and 10 μM of IAA and GA, respectively. Additionally, 1 μM PAC was set as the inhibitor of GA. After 5 days treatment, the shoot length of seedlings was measured to analyse the response to plant hormone.

### Shoot gravitropism assay

The gravitational response was measured according to the methods described previously (Zhang *et al*., 2018). Seeds of NILs and *GLW9* transgenic lines were cultured on 1/2 MS medium after dehulling and surface sterilization. Seeds were germinated and grown at 28°C for 6 days under a 16‐h light and 8‐h dark cycle. Subsequently, the seedlings were rotated 90° vertically and allowed to grow for an additional day. The degree of shoot curvature in the seedlings was recorded. The base of the curved seedling was sectioned along the central axis into upper and lower halves, and samples were collected separately for auxin content examination. At least 10 biological replicates were conducted on each line.

### Primers

Primers used in this study are listed in Supplemental Tables S2.

### Statistical analysis

Data are presented as means ±standard deviation. Significant differences between paired groups were analysed by two‐tailed *t*‐tests and multiple comparisons were assessed for significant differences by Duncan's tests (*P* < 0.05) using SPSS software.

## Funding

This work was supported by grants from the Ministry of Science and Technology (2021YFF1000200, 2022YFD1200100), National Natural Science Foundation of China (U21A20211), the fund of Hubei Science and Technology (2024BBA005) and the earmarked fund for the China Agriculture Research System (CARS‐01‐01).

## Conflict of interest

The authors declare they have no conflict of interest.

## Author contributions

Y.H. and A.Y. conceived the project; H.S. designed the research and analysed the data under the supervision of Y.H.; Y.Z. and P.Y. constructed the foundational materials; L.W., B.W., Y.W., G.L., Q.H., J.C. and J.L. assisted with part of experiments and data analysis; G.G., Q.Z. and J.X. contributed the reagents and experimental support; H.S. wrote the manuscript and P.L. and H.Z., improved it.

## Supporting information


**Figure S1** Comparison of lemma cell number between NILs.
**Figure S2** Construction of *GLW9* transgenic lines.
**Figure S3** Grain shape examination and SEM observation of transgenic lines.
**Figure S4** Cellular observation of the stem nodes.
**Figure S5** Comparison of yield related traits among NILs and *GLW9* transgenic lines.
**Figure S6** Structure of vectors used for analysing activation of GLW9 on expansin genes.
**Figure S7** Vector construction for regulation analysis of GLW9 on *EXPA6* promotor.
**Figure S8** Sequencing peak map of *expa6‐1/2*, *glw9‐1/2* and WT lines.
**Figure S9** Comparison of lemma cell number among WT and *expa6* lines.
**Figure S10** Comparison of plant hormone content between NILs.
**Figure S11** Expression analysis of genes response to IAA and GA in NILs.
**Figure S12** Root length after IAA treatment and shoot length after GA treatment.
**Figure S13** Vector construction for regulation analysis of GLW9 on *OsPIN1b* promotor.
**Table S1** Agronomic traits of NILs and transgenic plants.
**Table S2** Primers used in this study. [Correction added on 18 April 2025, after first online publication: The supporting information has been updated in this version.]

## Data Availability

The data that supports the findings of this study are available in the supplementary material of this article.
