# Supplementary material for: A DOF transcription factor GLW9/OsDOF25 regulates grain shape and tiller angle in rice
Source: Plant Biotechnol J. 2025 Mar 22;23(6):2367–82. doi: 10.1111/pbi.70064 (PMC12120872; doi:10.1111/pbi.70064)
Supplement: Supplementary file 1 — Figure S1 Comparison of lemma cell number between NILs. Figure S2 Construction of GLW9 transgenic lines. Figure S3 Grain shape examination and SEM observation of transgenic lines. Figure S4 Cellular observation of the stem nodes. Figure S5 Comparison of yield related traits among NILs and GLW9 transgenic lines. Figure S6 Structure of vectors used for analysing activation of GLW9 on expansin genes. Figure S7 Vector construction for regulation analysis of GLW9 on EXPA6 promotor. Figure S8 Sequencing peak map of expa6‐1/2, glw9‐1/2 and WT lines. Figure S9 Comparison of lemma cell number among WT and expa6 lines. Figure S10 Comparison of plant hormone content between NILs. Figure S11 Expression analysis of genes response to IAA and GA in NILs. Figure S12 Root length after IAA treatment and shoot length after GA treatment. Figure S13 Vector construction for regulation analysis of GLW9 on OsPIN1b promotor. Table S1 Agronomic traits of NILs and transgenic plants. Table S2 Primers used in this study. [Correction added on 18 April 2025, after first online publication: The supporting information has been updated in this version.] [file PBI-23-2367-s001.docx]

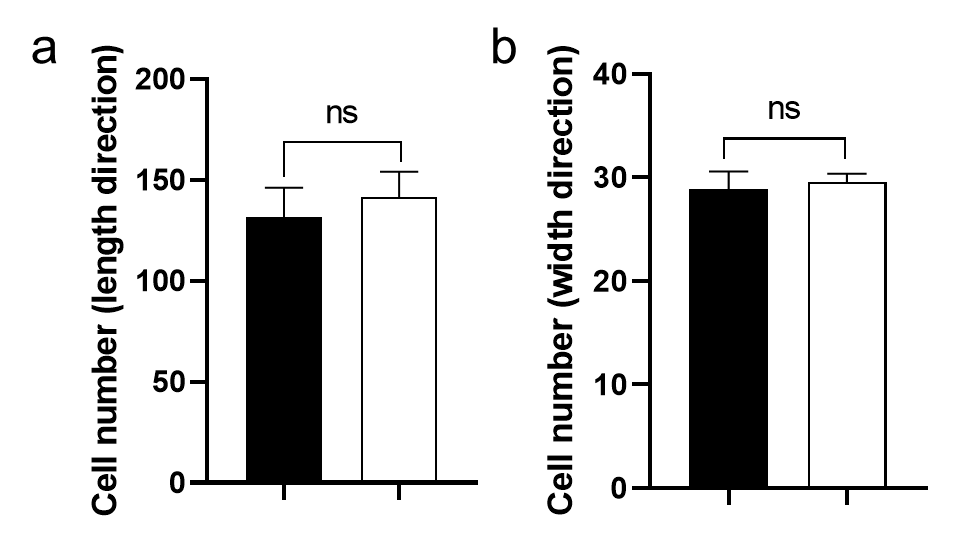


**Figure S1 Comparison of lemma cell number between NILs.** Significant differences were based on two-tailed *t*-tests. ns, not significant.


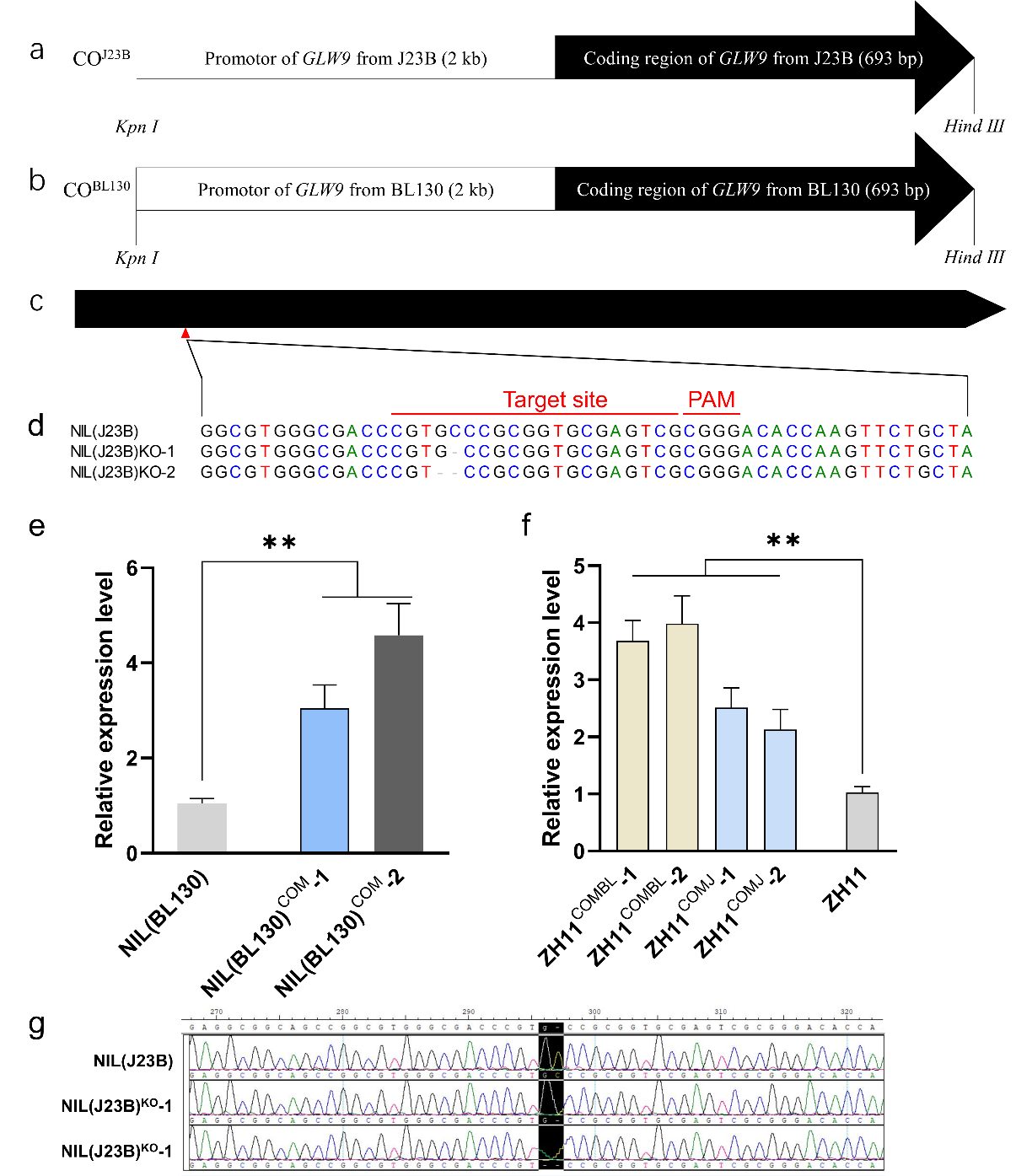


**Figure S2 Construction of *GLW9* transgenic lines. a-b** The structure of complementary vectors CO^J23B^ and CO^BL130^. **c-d** Sequence of the *GLW9* target site of NIL(J23B) and knockout lines. **e** Comparison of *GLW9* expression level among *GLW9* complementary transgenic lines and NIL(BL130). **f** Comparison of *GLW9* expression level among ZH11 and its complementary lines. Significant differences were based on two-tailed *t*-tests. **, *P* <0.01. **g** Sequencing peak map of NIL(J23B) and knock-out lines.


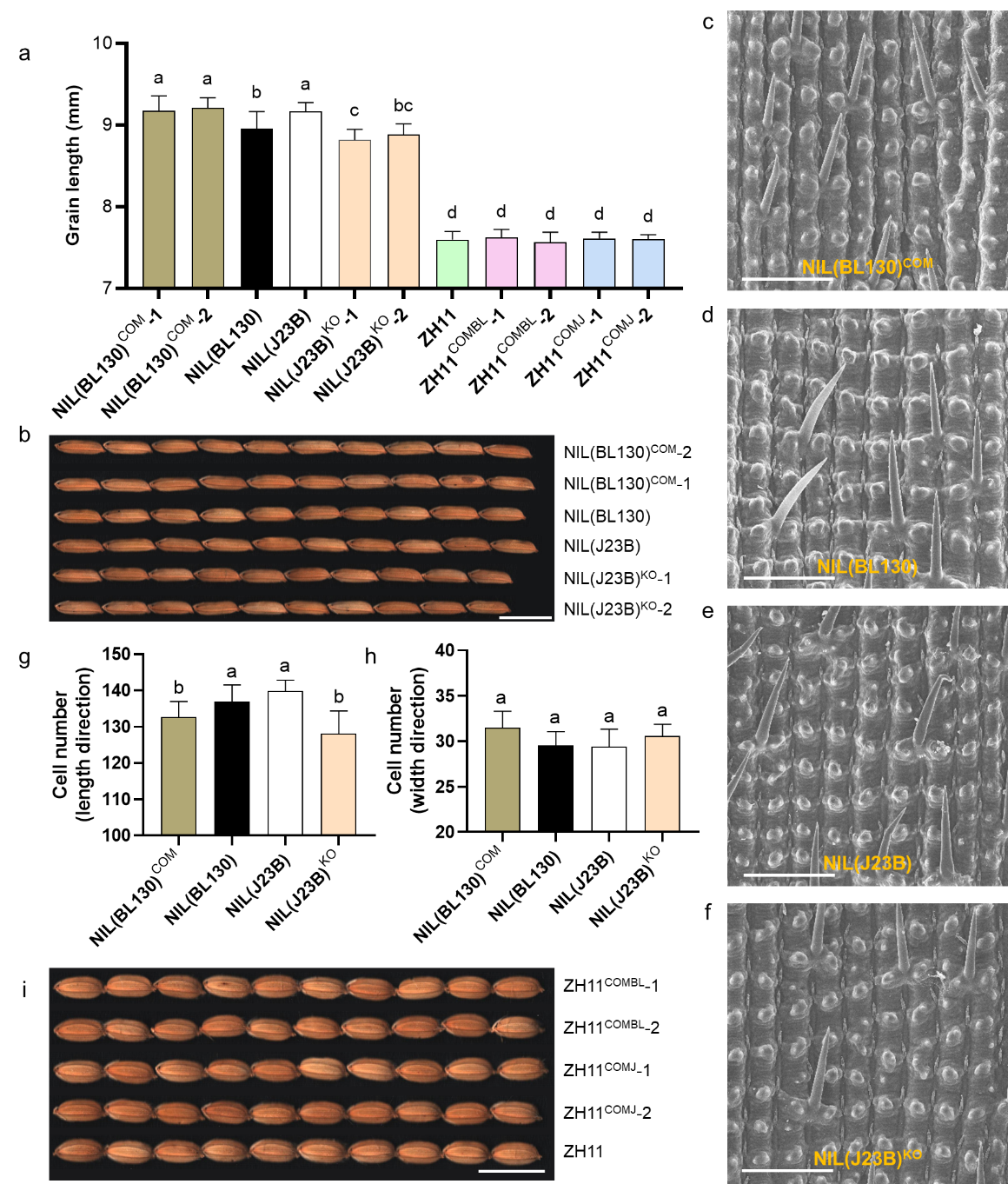


**Figure S3 Grain shape examination and SEM observation of transgenic lines. a-b** Comparison of GL among *GLW9* transgenic lines and background lines. Scale bar, 10 mm. Significance of differences was determined by Duncan’s multiple range test. **c-h** Comparison of cell size (**c-f**) and cell number (**g-h**) among *GLW9* transgenic lines and background lines. Scale bars, 200 μm. Significance of differences was determined by Duncan’s multiple range test. **i** Grain shape of ZH11 and complementary transgenic lines.


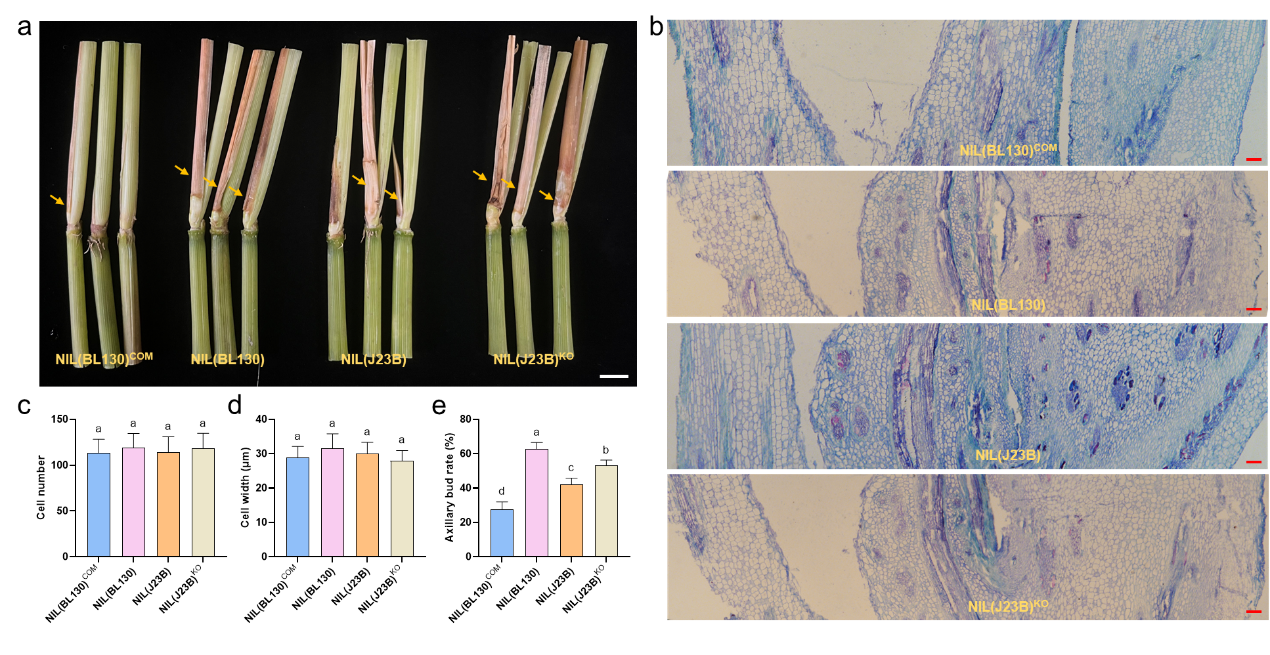


**Figure S4 Cellular observation of the stem nodes.** **a** The morphology of stem nodes among NILs and transgenic lines. The yellow arrows indicate the axillary buds. Scale bar, 1 cm. **b** Cell morphology observation of stem nodes among NILs and transgenic lines. Scale bars, 100μm. **c-d** Differences in cell number (**c**) and cell width (**d**) of stem nodes among NILs and transgenic lines. **e** Difference in axillary bud rate among NILs and transgenic lines. Significance of differences was determined by Duncan’s multiple range test.


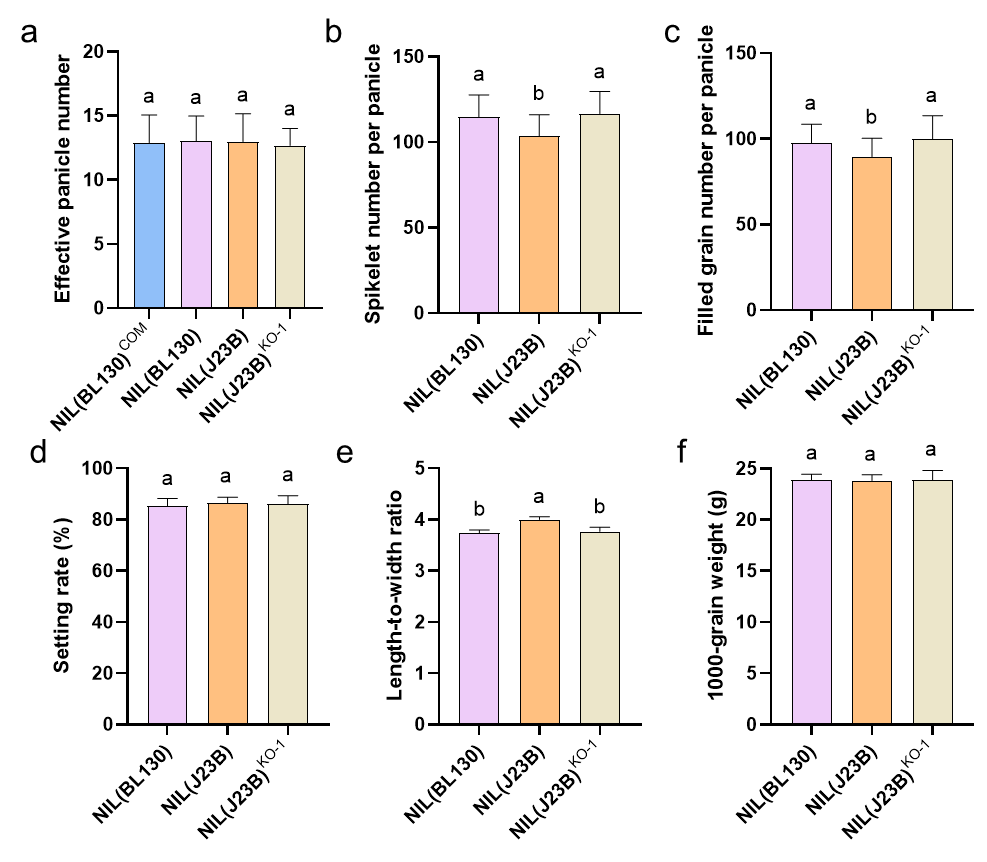


**Figure S5 Comparison of yield related traits among NILs and *GLW9* transgenic lines.** Significance of differences was determined by Duncan’s multiple range test.


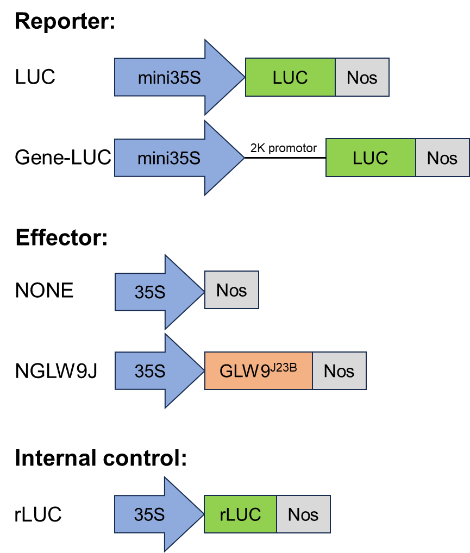


**Figure S6 Structure of vectors used for analyzing activation of GLW9 on expansin genes.**


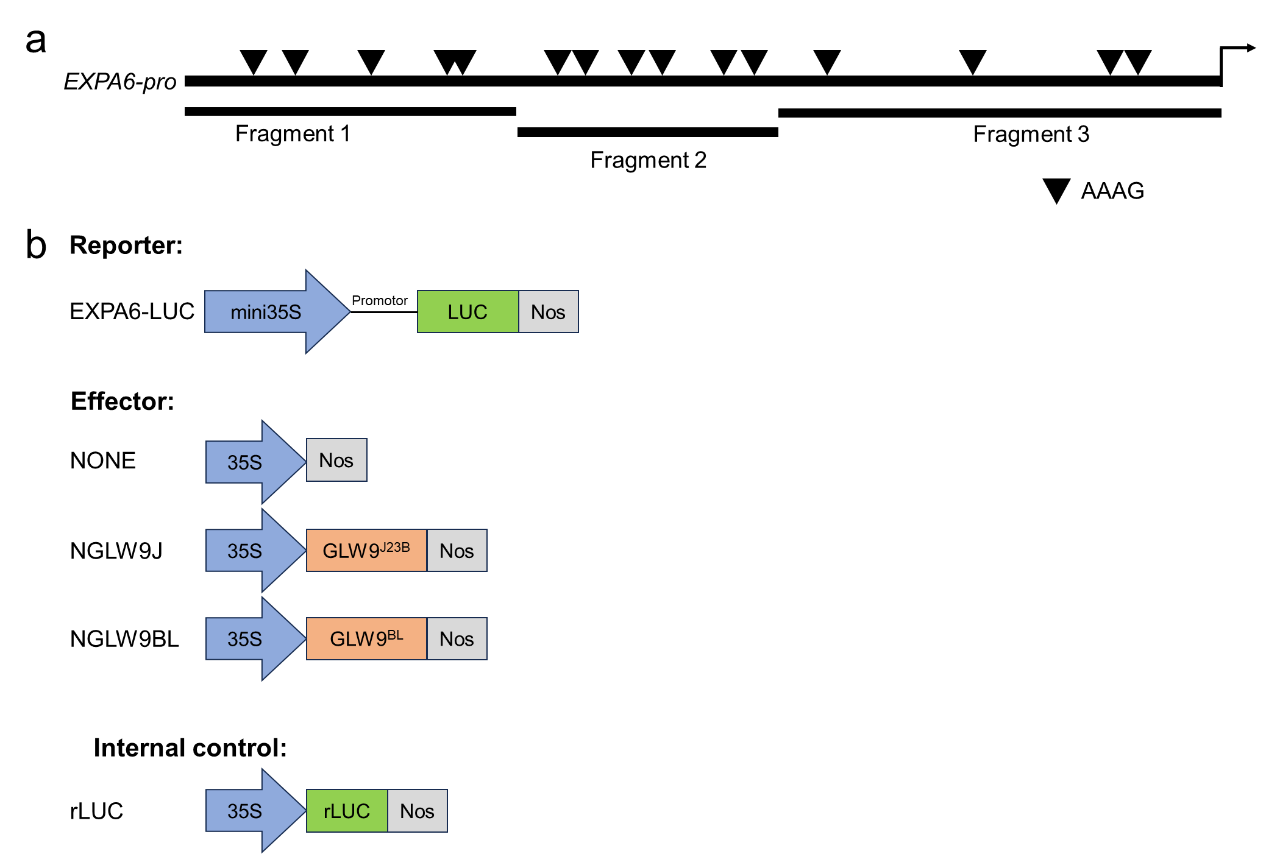


**Figure S7 Vector construction for regulation analysis of GLW9 on *EXPA6* promotor. a** CCCT sequences in *EXPA6* promotor. The arrow represents the direction of transcription. **b** Structure of vectors for regulation analysis of GLW9 on *EXPA6* promotor.

**
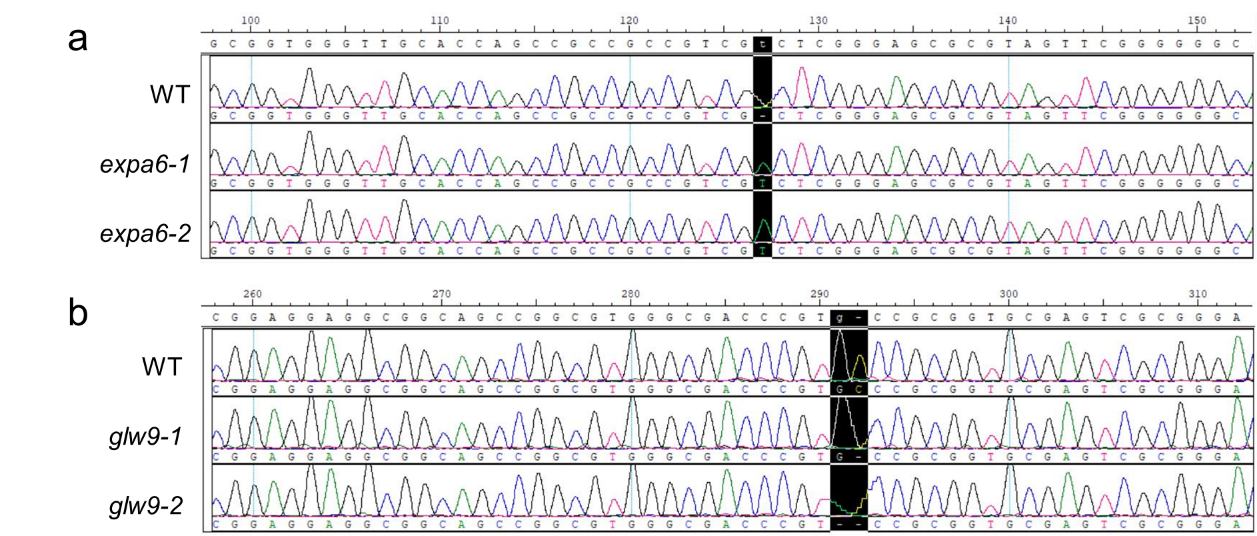
**

**Figure** **S8 Sequencing peak map of *expa6-1/2, glw9-1/2* and WT lines.**


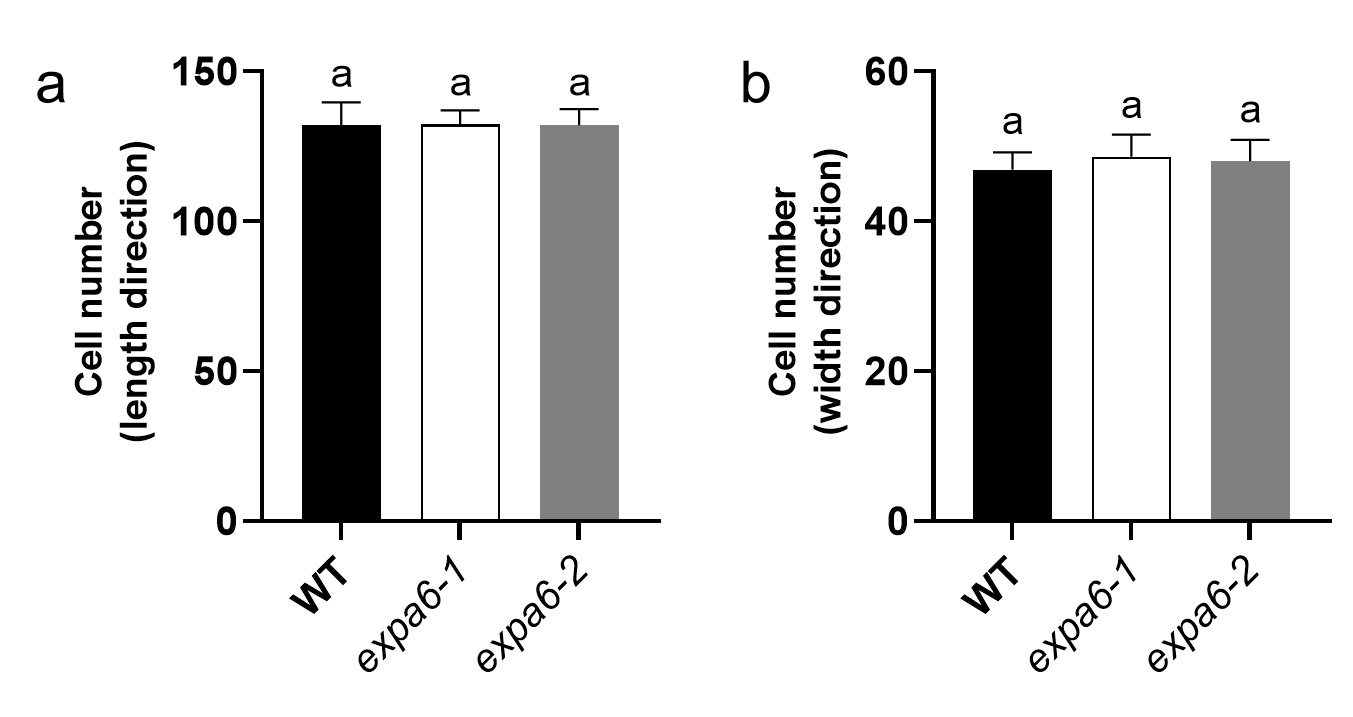


**Figure S9 Comparison of lemma cell number among WT and *expa6* lines.** Significance of differences was determined by Duncan’s multiple range test.


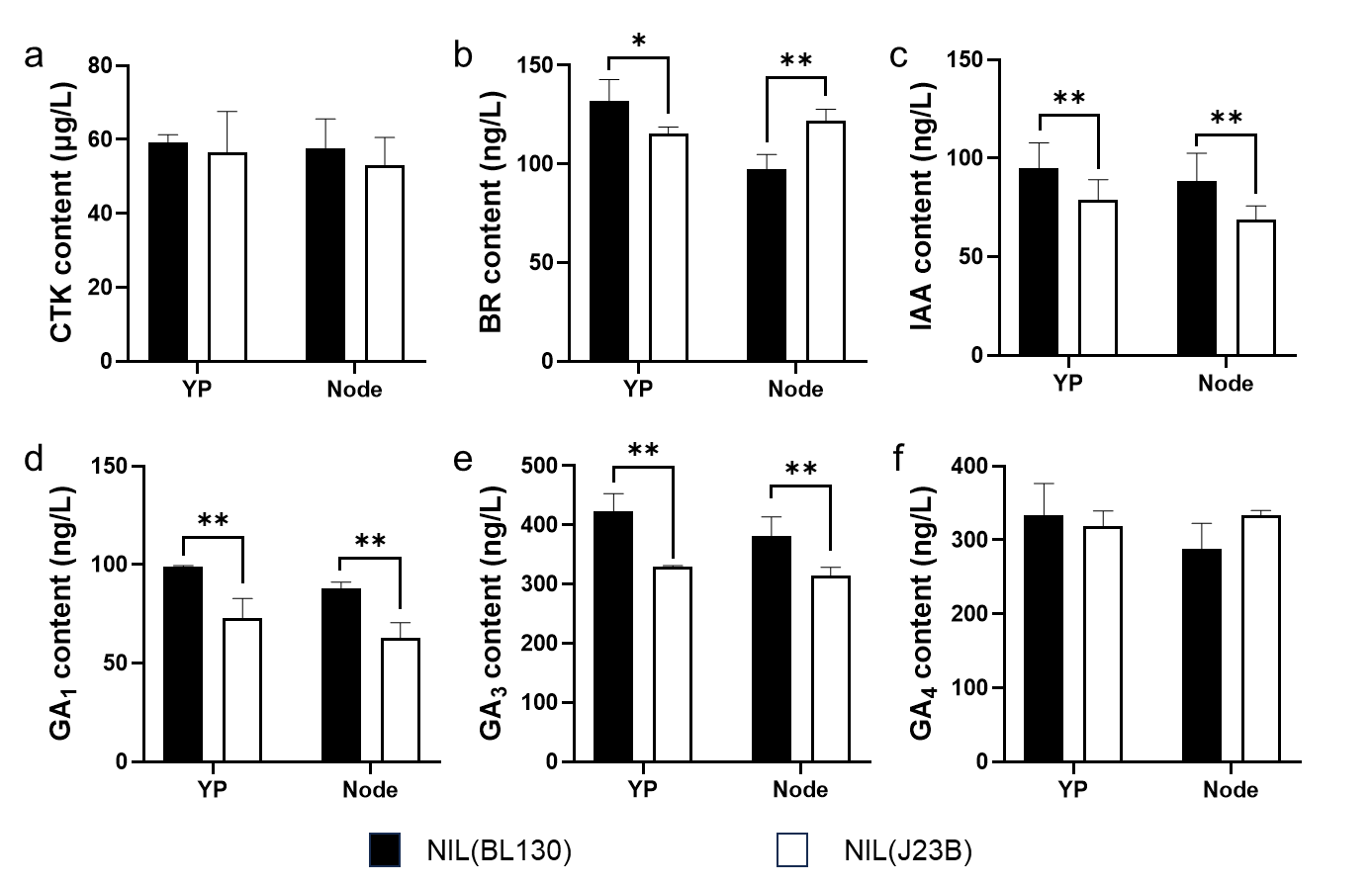


**Figure S10 Comparison of plant hormone content between NILs.** Significant differences were based on two-tailed *t*-tests. * and **, *P* <0.05 and *P* <0.01, respectively. YP, 5 cm young panicle. Node, basal internode.


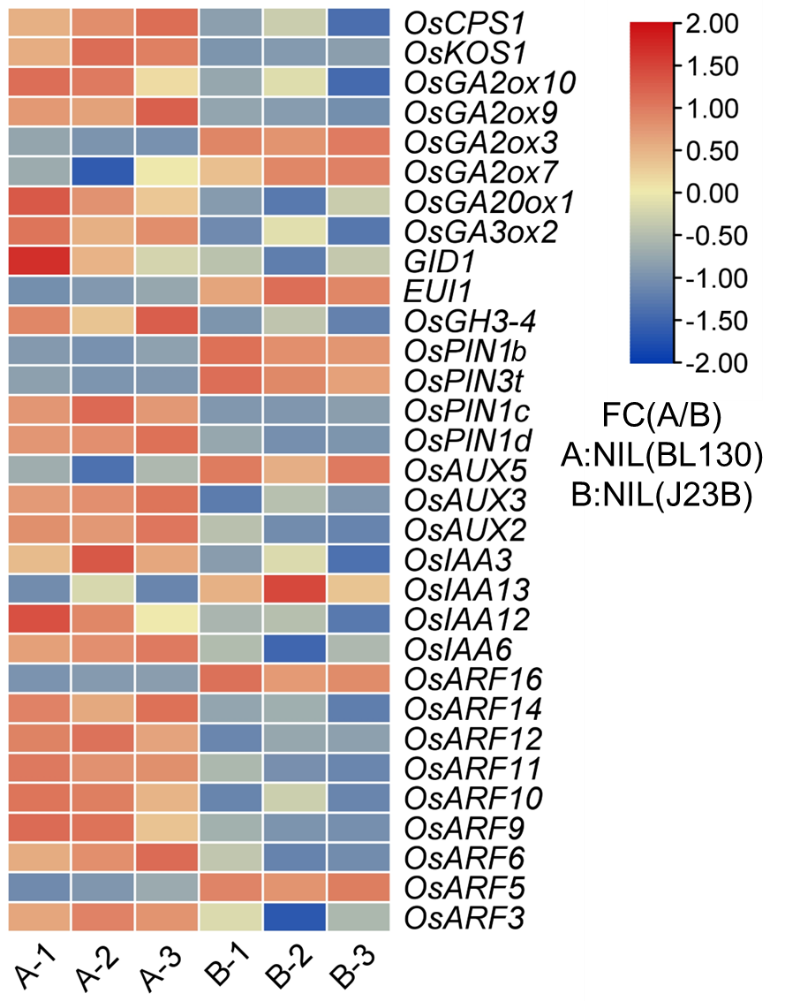


**Figure S11 Expression analysis of genes response to IAA and GA in NILs.**


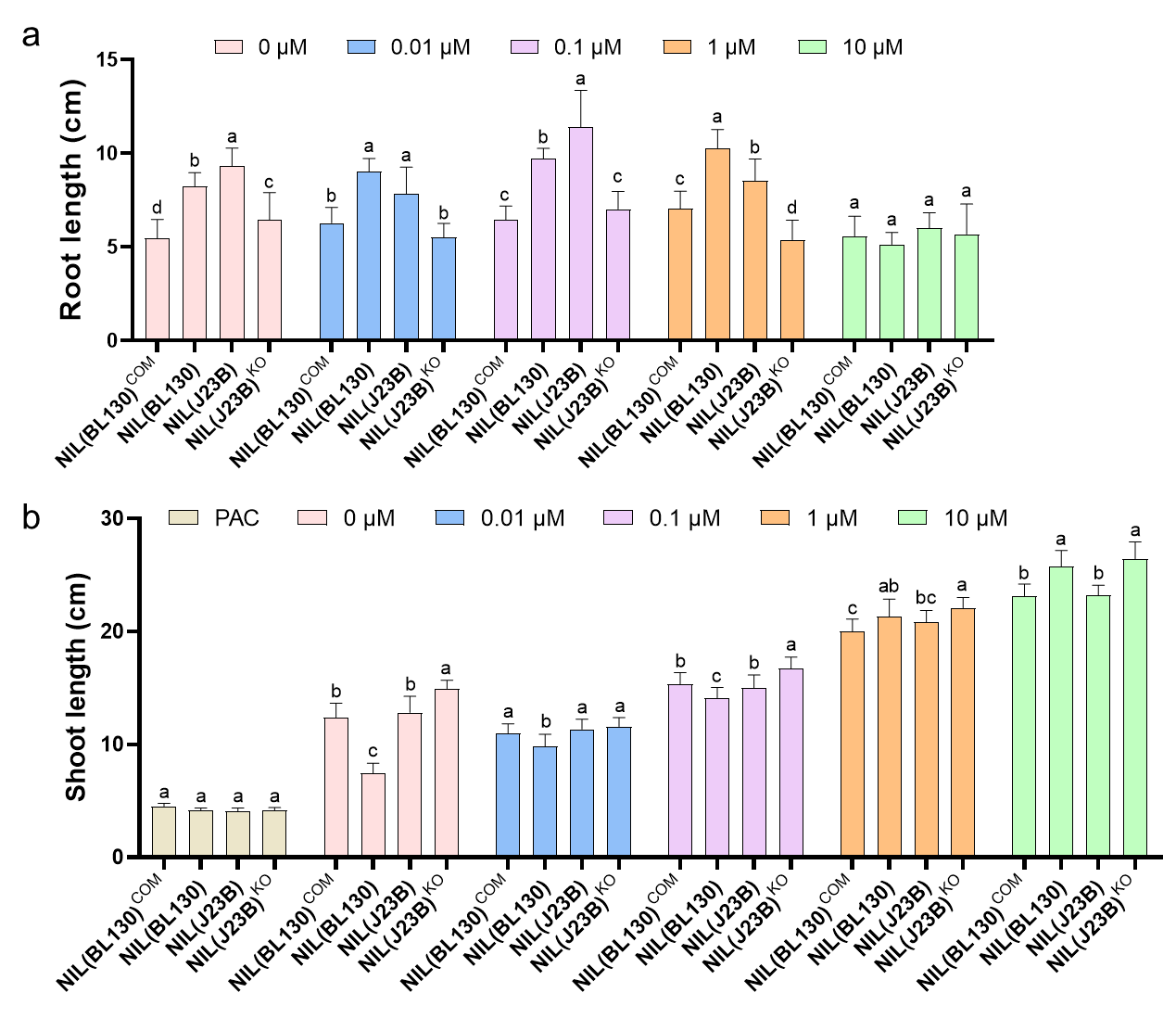


**Figure S12 Root length after IAA treatment and shoot length after GA treatment. a**  Comparison of root length among different lines after 7 days of IAA treatment. **b** Comparison of shoot length among different lines after 7 days of GA treatment. PAC was set as the inhibitor of GA. Significance of differences was determined by Duncan’s multiple range test.


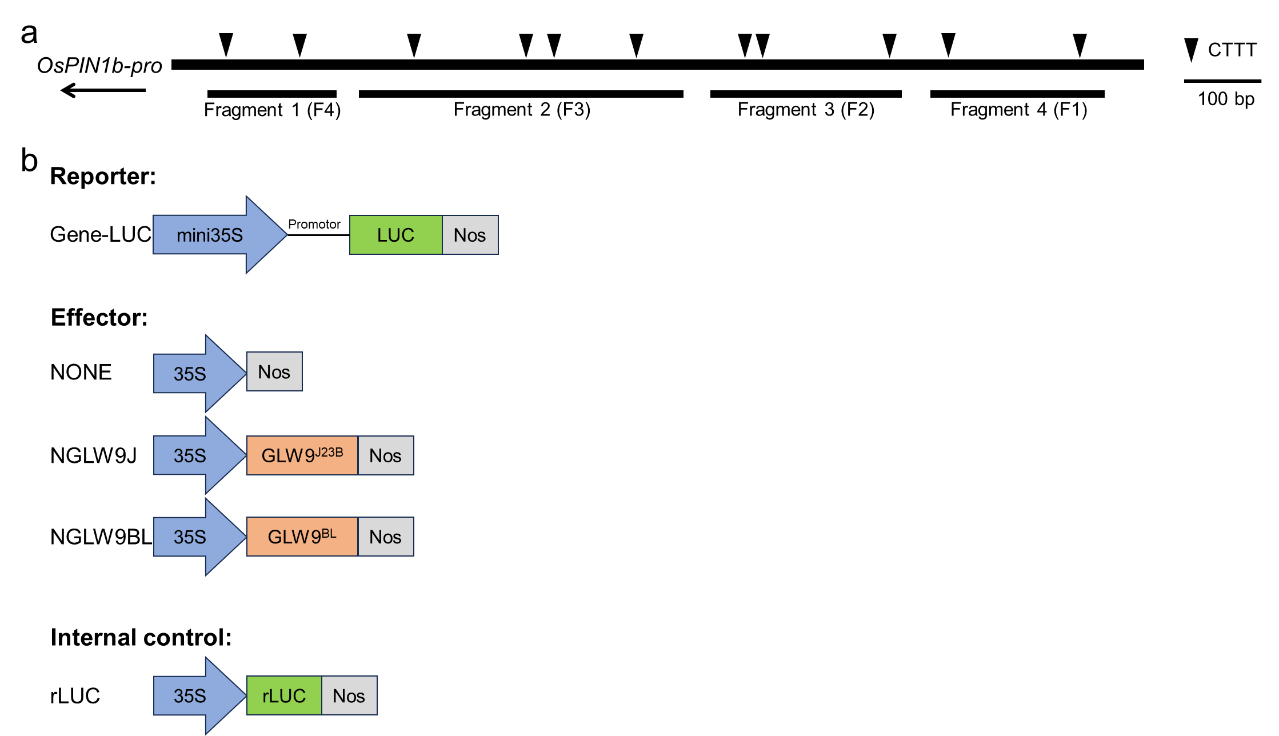


**Figure S13 Vector construction for regulation analysis of GLW9 on *OsPIN1b* promotor. a** AAAG sequences in *OsPIN1b* promotor. The arrow represents the direction of transcription. **b** Structure of vectors for regulation analysis of GLW9 on *OsPIN1b* promotor.

**Table S1 Agronomic traits of NILs and transgenic plants**

| Lines | *N* | Heading date (days) | Plant height (cm) | Effective panicle number | Spikelet number per panicle | Filled grain number per panicle | Setting rate (%) | Length-to-width ratio | 1000-grain weight (g) | Yield per plant (g) | Chalkiness rate (%) | Tiller angle (°) |
| --- | --- | --- | --- | --- | --- | --- | --- | --- | --- | --- | --- | --- |
| NIL(BL130) | 36 | 54.2±2.9a | 87.6±2.9b | 13.0±1.9a | 114.7±12.8a | 97.7±11.1a | 85.2±3.1a | 3.7±0.1b | 23.9±0.6a | 30.1±3.4a | 70.7±6.9a | 13.5±1.9a |
| NIL(J23B) | 36 | 54.3±3.0a | 89.3±3.0a | 13.0±2.2a | 103.6±12.3b | 89.6±10.8b | 86.5±2.3a | 4.0±0.1a | 23.8±0.6a | 27.7±2.8b | 47.4±5.8c | 9.3±2.1b |
| NIL(J23B)^KO^ | 36 | 54.0±3.0a | 90.0±3.0a | 12.7±1.3a | 114.7±13.1a | 99.9±13.7a | 86.1±3.3a | 3.8±0.1b | 23.9±1.0a | 30.1±4.9a | 55.1±4.1b | 12.0±1.8a |
| NIL(BL130)^COM^ | 36 | 55.1±2.5a | 90.1±2.5a | 12.9±2.0a | - | - | - | - | - | - | 52.1±3.8b | 8.9±0.9b |

*N*, number of plants. The letters following the numbers indicate the presence of significant differences. Significance of differences was determined by Duncan’s multiple range test.

**Table S2 Primers used in this study**

| Primer name | Forward sequence | Reverse sequence | Note |
| --- | --- | --- | --- |
| M22 | TCAGTGTGTCAGACTAAGGTTTTG | TTGTAGCAAGACCTGCTGCTT | Primers for fine-mapping |
| M23 | TCGTCAGAAAAGTGCAAGACA | AAAACCAACTACACAGTTCGACA |  |
| S1 | CATATGGCTCCCACGATCCC | GCACGGACTCCAAGACGTAA |  |
| S2 | TTACGTCTTGGAGTCCGTGC | TTGGAGTTCTTGGAGCGCTT |  |
| S3 | ACACCGTAGAAATGGCCGAG | AACCGAACGAACAGATCCCC |  |
| S4 | AGCTGAACTGTACTTGCGTGA | GAAGCGATTCCAAAAGCCCC |  |
| S5 | TCTCGTGCGAGTCGTTCAAA | CCGACAGATGCGGGCTATAG |  |
| S6 | GAGTAGGCAAGGGAGGGAGA | CACCGGAGATCCCTTAACCG |  |
| S7 | GTTTTCCGCACCCCCTTTTC | ACATATGCCGTAGTGCTCCG |  |
| S8 | CACTGTAAGCGAGACACCGT | GCATGTCATGTCCATGTGGC |  |
| GLW9-Sub | CACTGTTCCTTAACCTGCCAaagcttatggcgagtagcgaagac | acgacggccagtgccaagcttttaagcaccggtggagtgac | Primers for subcellular localization |
| GUS | CCAGGCAGTTTTAACGATCAGTTCGC | GAGTGAAGATCCCTTTCTTGTTACCG | Primers for genotype detection of transgenic plants |
| GLW9-seq | GTGAAAACACACCGTAGAAA | GCAGGTTAAGGAACAGTGAT |  |
| EXPA6-seq | CGTTGACGGTGAACCTGACG | AGATCGTCTAACTCACCACCT |  |
| PC1301-GLW9 | tacgaattcgagctcggtaccCACACGACTCACGAACAC | acgacggccagtgccaagcttTCATGGCAGGTTAAGGAACAG | Primers for vector construction of transgenic events |
| Cas-GLW9 | GCGTGGGCGACCCGTGCCCGgttttagagctagaaatagcaagtta | CGGGCACGGGTCGCCCACGCAACCTGAGCCTCAGCGCAGC |  |
| U6 | GCGTGGGCGACCCGTGCCCGgttttagagctagaaatagcaagtta | CGGGCACGGGTCGCCCACGCAACCTGAGCCTCAGCGCAGC |  |
| Cas-EXPA6 | GCTGCCACTGGCCGAGGCCGgttttagagctagaaatagcaagtta | CGGCCTCGGCCAGTGGCAGCAACCTGAGCCTCAGCGCAGC |  |
| RT-GLW9 | ACTTCCTCCTCCTCCTCCT | CGCTTGGAGTTCTTGGAG | Primers for quantitative RT-PCR |
| RT-EXP4 | AGCCTCAGCATAGCCCAAAA | TAGCACGGTGTCCTTCTTCG |  |
| RT-EXPA3 | TCAGAACTGCACCCAAGAGG | CTTCATTGTGCAGCTCGACG |  |
| RT-EXPA6 | ATGCCACATGCCCTGTACAA | TGTGGCTCCTGCAAGAGAAG |  |
| RT-EXPA7 | CGGAAGTTCTTGCCCTCGAA | CCAGAACTGGCAGTCCAACT |  |
| RT-EXPA10 | TCCTCCCCATCCAGTCCTTC | AGGATGCAGCCAAGAATCGA |  |
| RT-EXPB3 | AGCTGCTGTACTGGACGAAA | CTTCTCGCTCCGCATCACC |  |
| RT-EXPB5 | TACTACCTCGCCATCCTGGT | TTCCCGGTGTTGGAGTTGAG |  |
| RT-EXPLA1 | AGGCTCAAATTCGTCAGCGA | CGCCTTGGTAGAGGAAGCTG |  |
| RT-PIN1 | CGTTTTACAAGGGTCAGCGC | GGGGACACAGGATCCAGTTC |  |
| RT-Actin | GCTGACAGGATGAGCAAGGA | TTGGCAATCCACATCTGCTG |  |
| None-GLW9 | cccccgggctgcaggaattcATGCAGGAGGCGGGGCGACG | ataagcttgatatcgaattcTCATGGCAGGTTAAGGAACAG | Primers for vector construction of dual-luciferase assays |
| 190LUC-EXPB5 | taaaacgacggccagtgccaagcttCATTAAAAGAACACAACACGG | aggaagggtcttgcagatctTGCTATTTTCTTGTTGGGCA |  |
| 190LUC-EXPB3 | taaaacgacggccagtgccaagcttTGTTCCTGCTGAATCCTTTG | aggaagggtcttgcagatctAGCTGCCACCACTCCAACCA |  |
| 190LUC-EXPA10 | taaaacgacggccagtgccaagcttTTCACAAGTTACCACCGCCT | aggaagggtcttgcagatctGGCTCCAAAGGTGTTCGTCG |  |
| 190LUC-EXPA7 | taaaacgacggccagtgccaagcttGATTTAGTATTGCCAATACT | aggaagggtcttgcagatctTGTGAATGCGAGTGGCAACG |  |
| 190LUC-EXPA6 | taaaacgacggccagtgccaagcttTTAATTTCTGGTTCTAACCA | aggaagggtcttgcagatctTGGGGTAGTTTGGTGCGTTA |  |
| 190LUC-EXPA3 | taaaacgacggccagtgccaagcttCTGAAAGAAGAGCGGGCATT | aggaagggtcttgcagatctGCAAAAATTAATCTAGACAG |  |
| 190LUC-EXP4 | taaaacgacggccagtgccaagcttCATCACCAAATGCTGAGTCC | aggaagggtcttgcagatctGGCTGGTGATGGTGTCTAAA |  |
| 190LUC-EXPA6-F1 | taaaacgacggccagtgccaagcttCATGGCTGTAGCTGTAGGCC | aggaagggtcttgcagatctTCAACGACACCCATGATCCG |  |
| 190LUC-EXPA6-F2 | taaaacgacggccagtgccaagcttCGGATCATGGGTGTCGTTGA | aggaagggtcttgcagatctAGGTGGTGAGTTAGACGATCT |  |
| 190LUC-EXPA6-F3 | taaaacgacggccagtgccaagcttAGATCGTCTAACTCACCACCT | aggaagggtcttgcagatctTGGGGTAGTTTGGTGCGTTA |  |
| 190LUC-PIN1 | taaaacgacggccagtgccaagcttCACCGTCCGTAACCGAGATCC | aggaagggtcttgcagatctCTTCGCCCCCCCTCTTCCCT |  |
| 190LUC-PIN1-F1 | taaaacgacggccagtgccaagcttTTCAATAGTAGTACACCACG | aggaagggtcttgcagatctCAGCGCGGCCCGAGCTCGGT |  |
| 190LUC-PIN1-F2 | taaaacgacggccagtgccaagcttATAGCTGCAAGTTATTGCCA | aggaagggtcttgcagatctAGTGATGAGAGACATTGCTT |  |
| 190LUC-PIN1-F3 | taaaacgacggccagtgccaagcttAGCTGGGTGATCTATCTGGT | aggaagggtcttgcagatctCGTTGCTTGTGATTGTTCCA |  |
| 190LUC-PIN1-F4 | taaaacgacggccagtgccaagcttAACCGAGATCCAACGGCTGA | aggaagggtcttgcagatctTGCCACAAACTTCTCCATTT |  |
| GLW9-GST | TTCCAGGGGCCCCTGGGATCCATGCAGGAGGCGGGGCGACG | CTCGAGTCGACCCGGGAATTCTGGCAGGTTAAGGAACAGTG | Primers for EMSA and ChIP-qPCR |
| EXPA6-P1 | TCAGGAGTAGATAGCTCACA | AGAATTCACCCATTGCAA |  |
| EXPA6-P2 | TTGCAATGGGTGAATTCT | GACTCCTAATTGCAGTTCGTGA |  |
| EXPA6-P3 | TCACGAACTGCAATTAGGAGTC | GGTACGTCATCCTGAATCGTTT |  |
| EXPA6-P4 | AAACGATTCAGGATGACGTACC | CGGATCATGGGTGTCGTTGA |  |
| PIN1-P1 | CGTTGCTTGTGATTGTTCCA | ATGTCTGTGTTTGCATTTGC |  |
| PIN1-P2 | GCAAATGCAAACACAGACAT | AGCTGGGTGATCTATCTGGT |  |
| PIN1-P3 | CATTGCTTCCGGCGCTAGAC | ACGAGCTTCTGTACGTACCT |  |
| PIN1-P4 | AGGTACGTACAGAAGCTCGT | TGCAGGCTAGCACTAGCAGA |  |
| PIN1-P5 | TCTGCTAGTGCTAGCCTGCA | TAGCTGCAAGTTATTGCCAG |  |
